# Infrared Photodissociation Spectroscopy of Fluoride–Anion Hexafluoroisopropanol Complexes: Solvation-Suppressed Proton Transfer

**DOI:** 10.1021/acs.jpclett.5c00953

**Published:** 2025-07-07

**Authors:** Milena Barp, Florian Kreuter, Jiaye Jin, Ralf Tonner-Zech, Knut R. Asmis

**Affiliations:** Wilhelm-Ostwald-Institut für Physikalische und Theoretische Chemie, 9180 Universität Leipzig, Linnéstraße 2, 04103 Leipzig, Germany

## Abstract

We characterize the interaction in fluoride–anion complexes with up to three hexafluoroisopropanol (HFIP) molecules using gas-phase ion vibrational spectroscopy combined with electronic structure calculations and an energy decomposition analysis. Infrared photodissociation spectra (1000–3350 cm^–1^) of D_2_-tagged [F, (HFIP)_*n*_]^−^ complexes with *n* = 1–3 allow for a detailed characterization of the extent of charge transfer and hence the extent of proton transfer in these prototypical systems. The OH stretching frequency for *n* = 1 is observed at 1436 cm^–1^. This dramatic red-shift Δν_OH_ by more than 2230 cm^–1^ relative to that of free HFIP is associated with the presence of a very strong, single-well ionic hydrogen bond and, consequently, a shared proton motif in [F, HFIP]^−^. Δν_OH_ is reduced to 1878 cm^–1^ (*n* = 2) and 1067 cm^–1^ (*n* = 3) in the larger complexes, indicating a substantial weakening of the ionic hydrogen bond concomitant with a suppression of proton transfer to the fluoride anion upon microsolvation with multiple HFIP molecules. This pronounced dependence of fluoride’s hydrogen-bond acceptor ability on its direct solvation environment contributes to a better understanding of HFIP’s role in promoting fluorination reactions.

## Graphical abstract

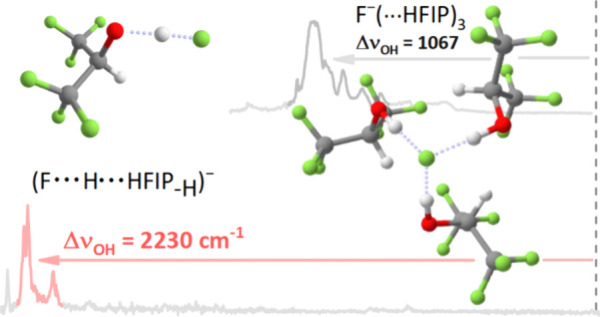

The selectivity and yield of fluorination reactions depend on a solvent’s ability to moderate the fluorinating reagents’ properties, typically by hydrogen bonding.^1–3^ Recently reported fluorination strategies are particularly efficient when 1,1,1,3,3,3-hexafluoroisopropanol (HFIP) is the reaction solvent.^3–5^ Indeed, HFIP’s strong hydrogen bond donor ability combined with its low nucleophilicity^6^ results in an enhanced reaction rate, a stronger activating effect, and less side reactions compared to other alcohols.^5^ Hence, a profound understanding of the coordination structures in fluoride/alcohol solutions is required.^1^ However, accurately modeling solution-phase systems remains challenging. In this context, studies on isolated model systems are relevant because they allow the dissection of individual intermolecular interactions in detail using affordable high-level electronic structure calculations.

While a free fluoride anion (F^–^), just like a free proton, does not exist in solution, it is stable in the gas phase. Its microsolvation can be studied using the highly sensitive and selective tool kit of gas-phase ion spectroscopy and by precisely controlling the number of added solvent molecules. Here, we use infrared photodissociation (IRPD) spectroscopy, complemented by electronic structure calculations, to study the hydrogen-bond nature in fluoride–anion complexes with up to three HFIP molecules.

Isolated halide–anion complexes with HFIP were originally studied by Wang and co-workers using anion photoelectron spectroscopy.^7^ While the spectra of Cl^–^, Br^–^, and I^–^ are indicative of the formation of a structure in which X^–^ interacts with (intact) HFIP via charge–dipole interactions, the spectrum of [F, HFIP]^−^ is more similar to that of the deprotonated HFIP anion ([HFIP_–H_]^−^), implying the formation of a (HFIP_–H_)^−^···HF complex via proton transfer. This extraordinary role of the fluoride anion in gas-phase halide–anion complexes is similar to that reported previously for the corresponding complexes with water^8–18^ and methanol.^19–21^ A subsequent IRPD study highlighted the role of charge transfer in X^–^(HFIP) complexes (X^–^ = Cl^–^, Br^–^, and I^–^), which is directly reflected in frequency red-shift Δν_OH_ of the OH stretching fundamental.^22^ Here, we present the results of the extended study on fluoride–HFIP complexes.

In Figure 1, we compare the IRPD spectra of D_2_-tagged [F, (HFIP)_*n*_]^−^ with *n* = 1–3 to the spectra of D_2_-tagged [HFIP_–H_]^−^, [OH, HFIP]^−^, [Cl, HFIP]^−^, and [Cl, (HFIP)_2_]^−^ in the spectral region from 1000 to 3350 cm^–1^. Peak positions and assignments are reported in Table S2, and spectral scans up to 4000 cm^–1^ for D_2_-tagged [HFIP_–H_]^−^, [F, HFIP]^−^, and [OH, HFIP]^−^ are shown in Figure S3. Only the [Cl, HFIP]^−^ spectrum has been reported and assigned previously.^22^ This spectral region can be divided into two parts: the region above 1450 cm^–1^, where we expect to observe bands associated with the fundamental excitation of the OH, CH, and DD stretching modes, and the region below 1450 cm^–1^, which contains the bands associated with the excitation of the other HFIP normal modes, i.e., the CO, CF, and CC stretching and COH bending modes. The first spectral region directly reports on hydrogen bond-induced red-shift Δν_OH_, but its assignment is often complicated due to pronounced anharmonic effects,^23^ which typically (but not always) play a less relevant role in the fingerprint spectral region.^22^

**Figure 1:**
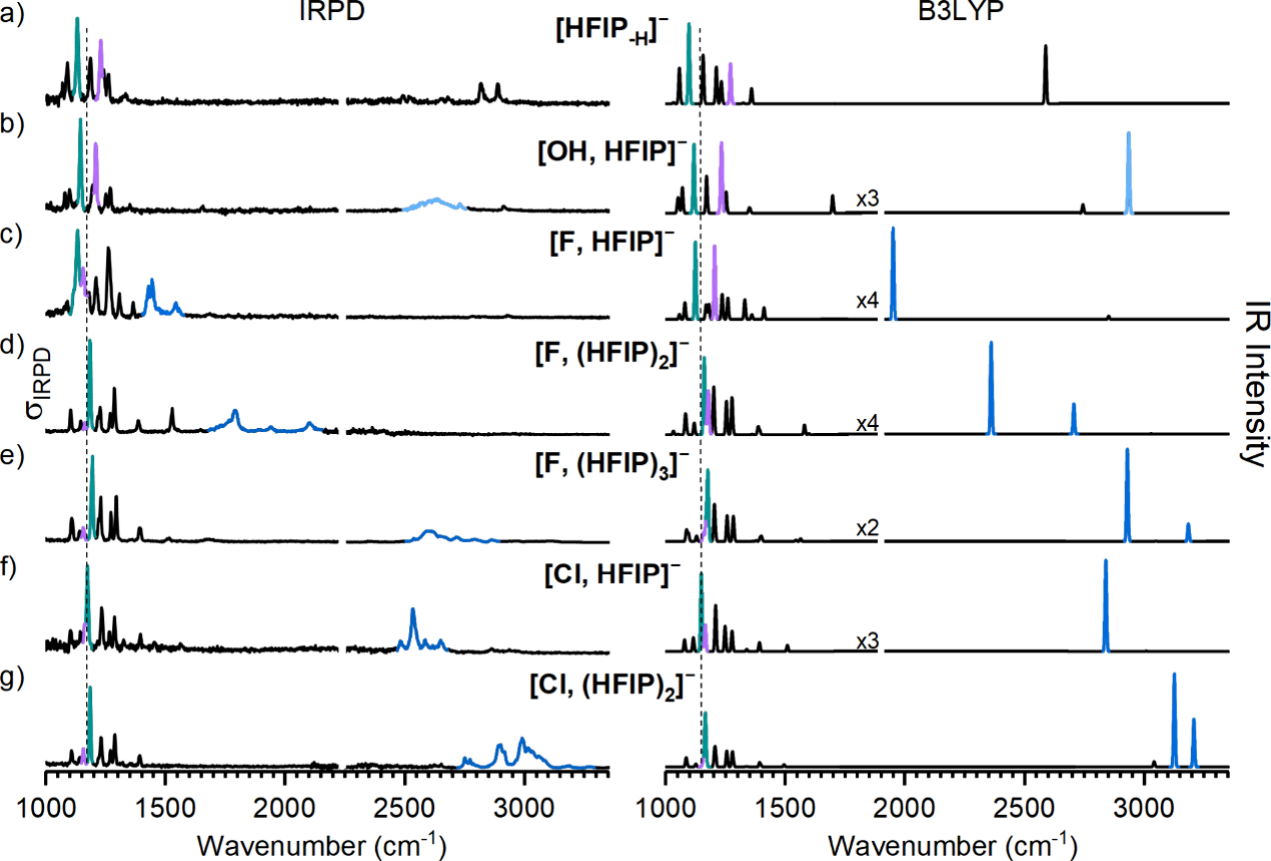
IRPD spectra of D_2_-tagged [HFIP_–H_]^−^, [OH, HFIP]^−^, [F, (HFIP)_1–3_]^−^, and [Cl, (HFIP)_1,2_]^−^ in the spectral range of 1000–3350 cm^–1^ recorded at 13 K (left) compared to harmonic IR spectra of the corresponding untagged complex (right) derived from B3LYP-D3­(BJ)/def2-TZVPP calculations. The dashed vertical line (1173 cm^–1^) is shown to guide the eye and coincides with the most intense CF stretching band (colored green) in the [Cl, HFIP]^−^ spectrum (see the text). The CO stretching band (lilac) and OH stretching features (blue) are also highlighted. The light blue color in panel b indicates the the OH stretching band of the OH group in H_2_O, rather than in the HFIP moiety (see the text for details). Note that each IRPD spectrum consists of two parts, separated by a gap at ∼2250 cm^–1^, which were measured with different IR laser configurations, complicating the comparison of the relative intensities between the two parts. See the Methods in the Supporting Information for experimental and computational details.

First, we focus on the spectral range below 1450 cm^–1^, where we expect bands that correspond to the vibrational transitions involving the HFIP backbone normal modes, which are characteristically different for intact versus deprotonated HFIP. The most intense band in this region is attributed to excitation of a CF stretching mode (see below) and is highlighted in green in Figure 1. In the [F, HFIP]^−^ spectrum (Figure 1c), it is observed at 1131 cm^–1^, while in the spectra of the larger complexes, it is found at a wavenumber that is more than 50 cm^–1^ higher, namely, for *n* = 2 at 1183 cm^–1^ (Figure 1d) and for *n* = 3 at 1194 cm^–1^ (Figure 1e). A closer look at all of the IRPD spectra in this region indicates that, indeed, the band position of the most intense band and the relative band intensities are similar for all of the spectra shown in Figure 1d–g, suggesting the presence of a similar HFIP motif. For the [Cl, HFIP]^−^ complex (Figure 1e), these bands were previously assigned to an intact HFIP moiety hydrogen-bonded to a halide anion (for band positions, see Table S2). In contrast, the [F, HFIP]^−^ spectrum in this region looks more similar to that of the deprotonated HFIP anion, which is shown in Figure 1a. This suggests a substantial degree of proton transfer from HFIP to the fluoride anion, in line with the larger anion proton affinity (PA) of F^–^ (PA = 1555 kJ mol^–1^)^24^ compared to that of (HFIP_–H_)^–^ (PA = 1443 kJ mol^–1^).^25^ Consequently, the proton transfer to the chloride anion (PA = 1395 kJ mol^–1^)^24^ is less pronounced. Further support comes from a comparison to the IRPD spectrum of [OH, HFIP]^−^ (Figure 1b), which exhibits a similar band pattern in this spectral region, in agreement with a deprotonated HFIP moiety as a result of the even larger PA of the hydroxyl anion (PA = 1633 kJ mol^–1^).^24^

Next, we focus on the bands above 1450 cm^–1^ and, in particular, those that are highlighted in blue in the spectra shown in Figure 1. These bands are assigned to excitation of HFIP’s hydrogen-bonded OH oscillator (see below). In the previously assigned [Cl, HFIP]^−^ spectrum (Figure 1f), the OH stretching fundamental is observed at 2535 cm^–1^.^22^ The three weaker peaks observed in the vicinity of the strong OH stretching fundamental were attributed to the excitation of two overtones and one combination transition, which gain intensity through a Fermi resonance, as reported previously. A comparable feature is observed in the spectrum of [F, (HFIP)_3_]^−^ at 2601 cm^–1^ (Figure 1e) and tentatively assigned accordingly, yielding a red-shift Δν_OH_ of 1067 cm^–1^. Note that the observed frequency, even at this small complex size, is in line with the OH band position in the infrared spectrum of a CsF/HFIP mixture.^1^ As the number of HFIP molecules in the fluoride complex is reduced, the center of this feature shifts to lower wavenumbers, namely, to 1790 cm^–1^ for *n* = 2 (Figure 1d) and to 1436 cm^–1^ for *n* = 1 (Figure 1c) and, consequently, Δν_OH_ increases to 1878 and 2232 cm^–1^, respectively.

The bands located at 1429 and 1443 cm^–1^ in the [F, HFIP]^−^ spectrum resemble those previously reported for the F^–^(H_2_O) complex.^11^ They were assigned to the excitation of the shared-proton motion in between the two heavy atoms in [F···H···OH]^−^. We assign them accordingly, confirming the presence of a similar motif in [F, HFIP]^−^. Interestingly, the lower-wavenumber bands (<1450 cm^–1^) in the [F, HFIP]^−^ IRPD spectrum are 50% broader (fwhm ∼ 15 cm^–1^) than those observed for the other complexes shown in Figure 1, suggesting that as proton transfer toward F^–^ proceeds, coupling between low-frequency modes involving the shared proton and HFIP “backbone” modes also increases, complicating the assignment of the spectra.

The observed red-shifts in Figure 1 confirm that indeed the extent of charge transfer is considerably larger in [F, HFIP]^−^ than in [Cl, HFIP]^−^, in agreement with the corresponding PAs. Moreover, the extent of charge transfer is largest in [OH, HFIP]^−^, where the corresponding O–H stretching feature involving HFIP’s O atom is shifted below the measurement window. Note that this system is better viewed as HO-H···[HFIP_–H_]^−^, and consequently, the band at 2632 cm^–1^ observed in Figure 1b is due to excitation of the hydrogen-bonded OH oscillator of the HOH moiety. The corresponding free O–H oscillator is observed at 3699 cm^–1^ (see Figure S3).

Moreover, for [Cl, (HFIP)_2_]^−^ (Figure 1g), the corresponding band is observed at 2991 cm^–1^ (Δν_OH_ = 677 cm^–1^), lending further support to the notion that Δν_OH_ decreases with an increasing number of HFIP molecules in the halide–anion complexes independent of the nature of the halide anion. Furthermore, the substantial decrease in Δν_OH_ indicates the formation of a symmetric solvation motif in which the next HFIP molecule adds to the halide anions’ first solvation shell rather than forming intersolvent hydrogen bonds. This agrees with HFIP’s strong hydrogen-bond donor and weak hydrogen-bond acceptor ability. Consequently, a particular complex with multiple HFIP molecules exhibits hydrogen bonds of similar strength, which grow weaker with an increase in the number of solvent molecules since the charge (resulting from charge transfer) is now distributed equally over multiple molecules.

The above-described microsolvation trends are confirmed by the results of density functional theory (DFT) calculations, which allow for some additional insights. The global minimum-energy structures (Figure 2) identified for the microsolvated halide–anion complexes confirm that the HFIP molecules exclusively form ionic hydrogen bonds (instead of intersolvent hydrogen bonds). Complexes with multiple HFIP molecules form higher-symmetry structures in which the HFIP molecules assume symmetry equivalent positions.

**Figure 2:**
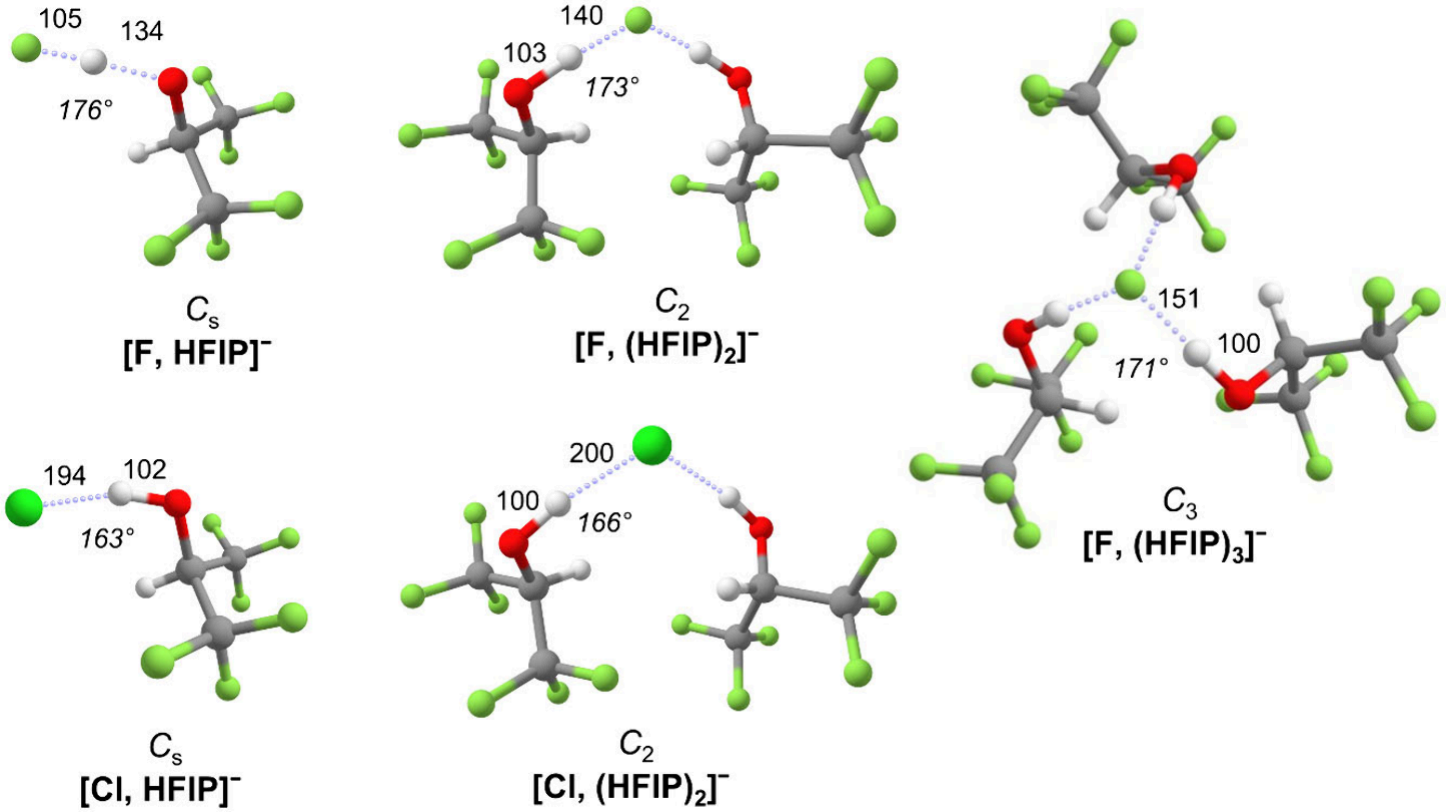
B3LYP-D3­(BJ)/def2-TZVPP minimum-energy structures, their symmetry, selected bond lengths (in picometers), and bond angles (in degrees) of the lowest-energy isomer for the halide–anion complexes [F, (HFIP)_1–3_]^−^ and [Cl, (HFIP)_1–2_]^−^. See the Supporting Information for computational details and Table S4 for additional structural parameters.

Of the halide complexes studied here, [F, HFIP]^−^ is the only system that exhibits a substantial degree of proton transfer, in agreement with the larger PA of F^–^ versus that of deprotonated HFIP (see above). The equilibrium F–H bond distance (*d*_e_^HF^ = 105 pm) is predicted to be substantially shorter than the O–H bond distance (*d*_e_^OH^ = 134 pm) but remains larger than that predicted for an isolated HF molecule (91.7 pm).^26^ In all of the other halide−anion systems, *d*_e_^OH^ remains close to 100 pm while *d*_e_^HX^ increases from 140 pm in [F, (HFIP)_2_]^−^ to 200 pm in [Cl, (HFIP)_2_]^−^. [F, HFIP]^−^ also exhibits the smallest heavy atom distance, confirming the well-documented trend that the relative hydrogen-bond strength in [X···H···O]^−^ complexes correlates with *d*_e_^OX^ (see Table S4).^27,28^ Finally, the ionic hydrogen bond is closest to linearity in [F, HFIP]^−^ (θ_OHX_ = 176°), and the hydrogen-bond angle θ_OHX_ decreases with an increase in *n* in the fluoride complexes. In contrast, θ_OHX_ is substantially smaller in [Cl, HFIP]^−^ (θ_OHX_ = 163°) as a consequence of a weaker secondary [Cl···H–C]^−^ hydrogen bond formed and also shows an opposing microsolvation trend.^22^

The predicted IR spectra, derived from unscaled harmonic DFT frequencies, of the minimum-energy structures are shown in the right panel of Figure 1. Satisfactory agreement between experimental and predicted spectra is observed in the region below 1450 cm^–1^, confirming our structural assignment (see section 5 of the Supporting Information for the calculated spectra of higher-energy isomers). The largest discrepancies are observed for the [F, HFIP]^−^ complex. We attribute this to the aforementioned pronounced anharmonic coupling between low-frequency modes and some of the HFIP “backbone” modes.^29^

The calculations also confirm that the position of the most intense CF stretching band in the spectra highlighted in green in Figure 1 indeed reports on the nature of HFIP in the complex; i.e., band positions above 1170 cm^–1^ indicate an intact HFIP moiety, while the formation of a substantially deprotonated HFIP unit is signaled by a band position up to 40 cm^–1^ below this value. A second reporter is the position of the CO stretching band, highlighted in lilac in Figure 1. Its position varies from 1157 cm^–1^ in the [Cl, (HFIP)_2_]^−^ spectrum, which contains the least perturbed HFIP molecules in the series studied here, and 1229 cm^–1^ in the [HFIP_–H_]^−^ spectrum, emphasizing that the CO bond strengthens and shortens as proton transfer proceeds (see Table S5 for geometrical parameters).

The agreement between the experimental and predicted IR spectra above 1450 cm^–1^ is considerably worse, as expected. The reason for this is twofold. First, the stretching mode associated with HFIP’s hydrogen-bonded hydroxyl group is anharmonically coupled with low-frequency modes, like the hydrogen bond and the heavy atom stretching modes of the [X···H···O]^−^ moiety, and this coupling increases with an increase in red-shift Δν_OH_. Second, the OH stretching fundamental can be involved in Fermi resonances with overtone and combination excitations, further complicating the spectral features and consequently increasing the discrepancies with regard to predictions based on the harmonic approximation.^10,11,22^

In order to obtain a better understanding of how the nature of the ionic hydrogen bond changes in each complex, we performed an energy decomposition analysis (EDA) (see section 1.2.3 of the Supporting Information for details of the method). The EDA results for the bond analysis in complexes involving Cl^–^, F^–^, and OH^–^ with HFIP are presented in Table 1. Notably, in the latter two cases, HFIP is predominantly deprotonated. Furthermore, hydrogen bonding in fluoride complexes with two HFIP molecules was also examined, as experimental findings indicate significant differences compared to complexes with only one HFIP molecule.

**Table 1:** EDA Results for Two Possible Fragmentation Channels of the [X, HFIP]^−^ Complexes (X = F, Cl, or OH)^a^

	[Cl, HFIP]^−^				[F, HFIP]^−^				[F, (HFIP)_2_]^−^		[OH, HFIP]^−^			
	D		I		D		I		I		D		I	
Δ*E*_int_	–440		–164		–186		–421		–169		–90		–621	
Δ*E*_int_(disp)^a^	–9	2%	–9	5%	–5	3%	–2	0%	–6	3%	–7	7%	–3	0%
Δ*E*_int_(elec)^a^	–431	98%	–156	95%	–181	97%	–419	100%	–163	97%	–84	93%	–618	100%
Δ*E*_Pauli_	617		111		223		420		154		116		544	
Δ*E*_elstat_ ^b^	–380	36%	–171	64%	–216	54%	–411	49%	–196	62%	–123	61%	–476	41%
Δ*E*_orb_ ^b^	–668	64%	–96	36%	–187	46%	–429	51%	–121	38%	–77	39%	–686	59%
Δ*E*_prep_	245		23		46		164		44		8		319	
*E*_bond_	–196		–142		–140		–257		–125		–82		–302	
*d*_e_(X–H)	102		193		131		107		141		158		102	

a Fragmentation leads to the formation of either deprotonated (HFIP_–H_)^–^ and HX or intact HFIP and X^–^, labeled D or I, respectively. The results for the I fragmentation channel of [F, (HFIP)_2_]^−^ are also listed. Energies are given in kilojoules per mole, and the bond distances are given in picometers.

b The percentage values give the relative contributions of dispersion and electronic effects to Δ*E*_int_.

c The percentage values give the relative contributions to the attractive EDA terms Δ*E*_elstat_ and Δ*E*_orb_.

Table 1 confirms that dispersion interactions are negligible (<10%). The results for two fragmentation channels leading either to deprotonated or intact HFIP, labeled D or I, respectively, are shown. Formation of intact HFIP is favored for [Cl, HFIP]^−^ due to the significantly lower interaction energy for channel I (−164 kJ mol^–1^) versus channel D (−440 kJ mol^–1^), suggesting a Cl^–^···H–OR motif. The interaction energy for channel I in [F, (HFIP)_2_]^−^ is similar (−169 kJ mol^–1^), and consequently, this complex contains two intact, hydrogen-bonded HFIP molecules as part of a F^–^(···H–OR)_2_ motif. In contrast, the situation is reversed for [OH, HFIP]^−^ (−90 kJ mol^–1^ for I vs −621 kJ mol^–1^ for D), which prefers a (HO–H···^–^OR) motif consisting of deprotonated HFIP hydrogen bonded to water. Note that the respective covalent bonds are not only stronger than the ionic hydrogen bonds but also characterized by a larger orbital contribution (∼60%), whereas the ionic hydrogen bonds are predominantly electrostatic (∼60%) in nature.

[F, HFIP]^−^ prefers a motif in which deprotonated HFIP is hydrogen-bonded to HF (−186 kJ mol^–1^ for I vs −421 kJ mol^–1^ for D). However, the difference between orbital and electrostatic interaction is substantially reduced for both channels. This suggests a more delocalized bonding interaction, in which the covalent and hydrogen-bond nature merge into one another, reminiscent of a strong, single-well hydrogen bond.^27,30–32^

To investigate the effect of this delocalized bonding interaction on the extent of delocalization of the shared proton in these strong hydrogen bonds qualitatively, we performed unrelaxed one-dimensional (1D) potential energy scans (see Figure 3 and section 1.2.2 of the Supporting Information for computational details) using the same computational method (MP2) as was reported in the previous study by McCoy and co-workers on [F, H_2_O]^−^, to allow a better comparison of the two sets of results.^11^ Note that the 1D-unrelaxed potential energy scans, or even the fully relaxed ones, are known to be insufficient to quantitatively reproduce the vibrational transition frequencies in shared proton systems. See refs 14 and 33 for recent work on higher-dimensional potentials and vibrational analysis of anion–water complexes.

**Figure 3:**
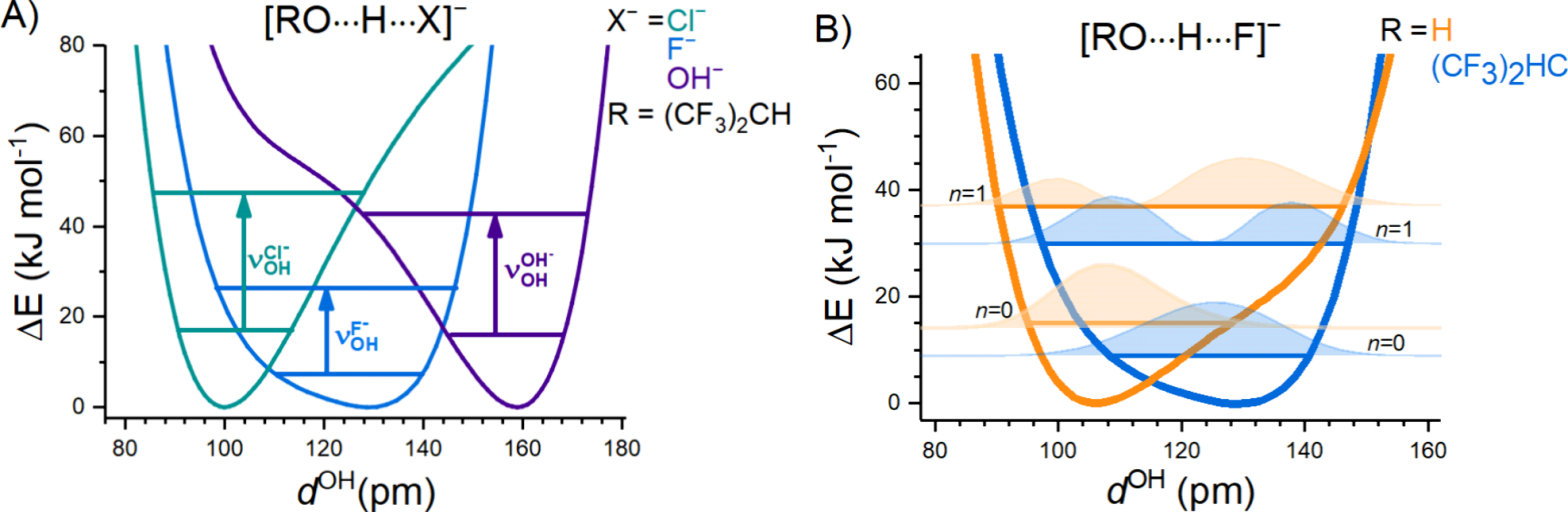
MP2/aug-cc-pVTZ 1D-unrelaxed potential energy scans of the RO–H distance (*d* ^OH^) in [Cl, HFIP]^−^ (green), [F, HFIP]^−^ (blue), and [HO, HFIP]^−^ (purple) (left, A) as well as [F, HFIP]^−^ (blue) and [F, HOH]^−^ (orange) (right, B). The arrows in panel A indicate the excitation of the 1 ← 0 transition. The shaded areas in panel B represent the probability density of the ground (*n* = 0) and first excited (*n* = 1) state for each complex.

The potentials obtained for [Cl, HFIP]^−^, [F, HFIP]^−^, and [OH, HFIP]^−^ are shown in Figure 3A, and the equilibrium RO–H distance (*d*_e_^OH^) is given in Table S6. The potentials reveal the qualitatively different nature of the three systems as a result of the difference in the respective anion PA, as discussed previously. This is reflected in the different values of *d*_e_^OH^ for Cl^–^ (101 pm), F^–^ (130 pm), and OH^–^ (165 pm), which correspond to a complex with intact HFIP in [Cl, HFIP]^−^ and a complex with deprotonated HFIP in [OH, HFIP]^−^. The [F, HFIP]^−^ complex exhibits an intermediate equilibrium structure, in which the (classical) proton is predicted to be closer to the center between the two heavy atoms. Furthermore, the potentials for [Cl, HFIP]^−^ and [OH, HFIP]^−^ are markedly asymmetric, exhibiting a shoulder at higher energies, while the [F, HFIP]^−^ potential is more symmetric and substantially broader at lower energies than the other two. This raises the interesting question of how the quantum nature of the proton influences its position.^34^

The expectation values of *d* ^OH^ in the vibrational ground state (⟨*d*_0_^OH^⟩) as well as in the first five vibrational excited states (⟨*d*_1–5_^OH^⟩) are listed in Table S6. For [F, HFIP]^−^, ⟨*d*_0_^OH^⟩ is 126 pm, slightly smaller than *d*_e_^OH^, and decreases within increasing vibrational level number, converging toward the center position of *d*_e_^OX^/2 = 118 pm, characteristic of an equally shared proton motif. As a result of the broader potential, this system also exhibits the smallest transition energy to the lowest vibrationally excited state. The other two systems show a more pronounced change in the expected value of ⟨*d*_i_^OH^⟩ with an increase in vibrational level number. While the density probability remains localized near one of the two heavy atoms in the vibrational ground state, it shifts toward the center position in the third (OH^–^) and fifth excited states (Cl^–^), where the respective potentials become significantly broader.

The 1D potential of [F, HFIP]^−^ is compared to that of [F, H_2_O]^−^ in Figure 3B. The latter potential exhibits the characteristic “shelf” structure that is responsible for “vibrationally induced” proton transfer, since the equally shared proton motif is only accessible in higher vibrationally excited states.^11^ In more detail, the ground state wave function (see Figure 3B) is localized closer to the hydroxyl anion and farther from the fluoride anion, while the situation is reversed in the first excited state. In contrast, the vibrational wave functions for [F, HFIP]^−^ are more symmetric, and consequently, this system does not exhibit “vibrationally induced” proton transfer but rather a nearly equally shared proton motif in both the ground and first excited states.

Finally, we discuss the influence of more than one solvent molecule in the X^–^(HY)_*n*_ complexes on observed vibrational red-shift Δν_OH_. For all of the systems listed in Table 2, we observed a decrease in Δν_OH_ with an increase in the number of solvent molecules *n*. The extent of this decrease in Δν_OH_ is different for each solvent molecule and depends on HY’s proton–donor ability. For binary fluoride–anion complexes F^–^(HY), which exhibit substantial proton transfer (HY = H_2_O or HFIP), the consequence of increasing the number of solvent molecules is more significant. Already for *n* = 2, proton transfer is substantially reduced, and for larger values of *n*, the increase in the degree of microsolvation leads to inhibition of proton transfer in these model systems, because a single strong hydrogen bond is replaced by multiple weaker ones.

**Table 2:** Vibrational Frequencies ν_OH_ (in cm^–1^) and Corresponding Vibrational Frequency Red-Shifts Δν_OH_ of Microsolvated X^–^(HY)_*n*_ Complexes with X^–^ = F^–^ or Cl^–^ and HY = HFIP, H_2_O, or CH_3_OH^a^

		HFIP		H_2_O		CH_3_OH	
anion	*n*	ν_OH_	Δν_OH_ ^a^	ν_OH_	Δν_OH_ ^a^	ν_OH_	Δν_OH_ ^a^
F^–^	1	1436	2232	1523^b^	2184	–	
	2	1790	1878	2520^c^	1187	–	
	3	2601	1067	2890^d^	817	3074^f^	607
Cl^–^	1	2535	1133	3158^e^	549	3162^g^	519
	2	2991	677	3130^c^	577	3241^g^	440

a Red-shift Δν_OH_ = free ν_OH_ – bonded ν_OH_, where the free ν_OH_ values are 3668 cm^–1^ (HFIP),^35^ 3707 cm^–1^ (H_2_O),^36^ and 3681 cm^–1^ (CH_3_OH).^37^

b From ref 10.

c From ref 18.

d From ref 17.

e From ref 22.

f From ref 19.

g From ref 38.

The present study on fluoride–anion complexes with HFIP sheds new light on the ionic hydrogen bonds in these isolated model systems. While the weaker ionic hydrogen bonds in the larger systems are predominantly electrostatic in nature, the stronger hydrogen-bond interaction in (F···H···HFIP_–H_)^−^, which exhibits an almost equally shared proton motif, gains substantially in covalent character. The addition of a second HFIP molecule is sufficient to suppress proton transfer, and even weaker hydrogen-bond interactions are expected in the larger complexes. The pronounced dependence of fluoride’s hydrogen-bond acceptor ability on its direct solvation environment reflects how HFIP moderates the properties of solutes in complex solutions. Furthermore, experiments on larger microsolvated complexes, particularly those considering HFIP/H_2_O mixtures, can yield important benchmark data for reliable solvation models in order to ultimately unravel HFIP’s unique role in fluorination reactions.

## Supplementary Material





## Data Availability

The data supporting this article have been included as part of the Supporting Information. Additional computational data can be found at https://zenodo.org/records/14894922.

## Notes

Methods (section 1), NaX mass spectra (section 2), IRPD spectra (section 3), geometrical parameters (section 4), higher-energy isomers (section 5), expected values for the OH position for vibrational levels 0–5 (section 6), [F, i-PrOH]^−^ complex (section 7), table of Δν_OH_ and ΔPA values (section 8), and additional references (section 9) (PDF)
Transparent Peer Review report available (PDF)
