# Supplementary material for: Infrared Photodissociation Spectroscopy of Fluoride–Anion Hexafluoroisopropanol Complexes: Solvation-Suppressed Proton Transfer
Source: J Phys Chem Lett. 2025 Jul 7;16(28):7187–93. doi: 10.1021/acs.jpclett.5c00953 (PMC12278305; doi:10.1021/acs.jpclett.5c00953)
Supplement: Supplementary file 1 [file jz5c00953_si_001.pdf]

Supporting Information

Infrared Photodissociation Spectroscopy of Fluoride Anion Hexafluoroisopropanol Complexes: Solvation-suppressed Proton Transfer

Milena Barp, Florian Kreuter, Jiaye Jin, Ralf Tonner-Zech, Knut R. Asmis**

Wilhelm-Ostwald-Institut für Physikalische und Theoretische Chemie, Universität Leipzig, Linnéstraße 2, 04103 Leipzig (Germany)

*E-mail: ralf.tonner@uni-leipzig.de, knut.asmis@uni-leipzig.de

Table of Contents

1. Methods 2
1.1 Experimental Methods 2
1.2 Computational Methods 3
1.2.1 Conformer Search and Vibrational Spectra 3
1.2.2 Potential Energy Scans 3
1.2.3 Energy Decomposition Analysis 4
2. NaX Mass Spectra 5
3. IRPD Spectra 6
3.1 Band Position 6
3.2 MP2 vs B3LYP 7
3.3 Extended Spectral Region (2250 cm^{-1} to 4000 cm^{-1}) 8
4. Geometrical Parameters 10
5. Higher Energy Isomers 11
5.1 $[F, (HFIP)_2]^-$ 11
5.2 $[F, (HFIP)_3]^-$ 12
5.3 $[Cl, (HFIP)_2]^-$ 13
6. Expectation Values for the OH Position for Vibrational Levels 0 to 5 14
7. $[F, i\text{-PrOH}]^-$ complex 16
7.1 IRPD spectrum of $[F, i\text{-PrOH}]^-$ complex 16
7.2 Energy Decomposition Analysis for $[F, i\text{-PrOH}]^-$ 17
8. Table $\Delta\boldsymbol{\nu OH}$ and $\Delta\mathbf{PA}$ 17
9. References 18

1. Methods

1.1 Experimental Methods

IRPD spectroscopic experiments were performed using a cryogenically cooled ion trap triple mass spectrometer described in detail elsewhere[1] and shortly here. Anion-molecule complexes are produced in a nanospray ion source from 0.5 mM sodium halide (NaF: Sigma Aldrich, 98%, NaCl) or sodium hydroxide and 0.5 mM HFIP solutions in MeOH/H_2O (1:2, v/v), see Fig. S1 for a typical mass spectrum of these solutions. The generated beam of anions is skimmed, collimated in a He-filled radio frequency (RF) octopole ion guide, mass-selected using a quadrupole mass filter and accumulated in a RF ring-electrode ion trap, held at a temperature of 13 K. To allow for continuous ion loading and ion thermalization, the trap is continuously filled with D_2 gas (AirLiquid, 99,8%). Many collisions of the trapped ions with the buffer gas provide gentle cooling of the internal degrees of freedom close to the ambient temperature. Ion–messenger complexes are formed via three-body collisions at sufficiently low ion-trap temperature.[2] Every 100 ms, all ions are extracted from the ion trap and focused, both spatially and temporally, into the centre of the extraction region of the orthogonally-mounted double-focussing reflectron time-of-flight (TOF) tandem mass spectrometer and detected using the background-free IR^1MS^2 detection scheme.[3] To this end, the ion packet is accelerated into the reflectron stage, ions spread out in space according to their mass-to-charge ratio (*m/z*) and are refocused at the initial extraction region. Prior to reacceleration towards the MCP detector, ions with a particular *m/z* value are irradiated by a properly timed and widely wavelength tunable IR laser pulse (bandwidth: 3.5 cm^{-1}). The IR pulse is supplied by an optical parametric oscillator/amplifier (LaserVision: OPO/OPA/$AgGaSe_2$) laser system pumped by an unseeded Nd:YAG laser (Continuum Surelite EX).[4] IRPD spectra are recorded by monitoring the population of the irradiated ions (and their photofragments) while the laser wavelength is monitored online using a HighFinesse WS6-600 wavelength meter. The wavelength is scanned continuously with a scan speed such that an averaged TOF mass spectrum (over 40 laser shots) is obtained every 2 cm^{-1}. Typically, three to five scans are measured and averaged and the photodissociation cross-section, σ_{IRPD}, is determined as described previously.[1,5]

The covered spectral range 950–4000 cm^{-1} requires the use of two different laser configurations, which differ in the laser beam/ion cloud overlap. This overlap difference is difficult to quantify and we therefore did not correct for this. Consequently, even though the signal is corrected for the wavelength-dependent laser pulse energy to obtain σ_{IRPD}, the individual spectra covering these two

spectral regions (950–2220 cm^{-1} and 2060–4000 cm^{-1}) may systematically differ in σ_{IRPD}. This should be considered, when comparing the relative IRPD band intensities between the two spectral regions.

1.2 Computational Methods

1.2.1 Conformer Search and Vibrational Spectra

For complexes with more than one HFIP molecule, conformer searches were performed using chemical intuition and the new global optimizer algorithm (GOAT) implemented in ORCA 6.0.[6–9] Geometries within 100 kJ mol^{-1} were further optimized using the Gaussian 16 rev. C01 package,[10] with the B3LYP functional[11] in combination with the third generation of Grimme's dispersion correction and Becke–Johnson damping (GD3(BJ))[12,13] and the def2-TZVPP[14] basis set. Geometry optimizations were followed by vibrational frequency calculations. For the binary complexes, calculations were also performed using the second-order Møller–Plesset perturbation theory (MP2)[15–18] in combination with the aug-cc-pVTZ Dunning basis set.[19,20] Simulated IR spectra are obtained by convolution with a Gaussian line-shape function with a full width at half maximum (FWHM) of 8 cm^{-1}.

1.2.2 Potential Energy Scans

The Gaussian 16 rev. C01 program package was also used to obtain one-dimensional unrelaxed scans along the O–H distance, d_{OH}, for all binary complexes using the method MP2 and the aug-cc-pVTZ basis set. The scanning parameters are reported in Table S1. The vibrational levels and transitions were retrieved from the eigenvalues of the solution of the vibrational Schrödinger equation. The potential was fitted by an 8th-degree polynomial and the wavefuncion Ψ_i for each vibrational level was constructed from a basis set of Gaussian functions from -0.8 to 1.2 Bohr spaced by 0.04 Bohr.The width of functions was 200 $Bohr^{-1}$.

Table S1. Scanning parameters for the construction of PES profiles, namely d_{OH} range and steps, both presented in pm.

	**From**	**To**	**Step**
**HFIP, F^-**	73.4	169.0	0.8
**HFIP, Cl^-**	28.3	271.3	1
**HFIP, OH^-**	64.9	185.9	1
**H_2O, F^-**	60.6	211.6	1

Relevant optimized geometries and frequency calculation outputs are shared on Zenodo at 10.5281/zenodo.14894922.

1.2.3 Energy Decomposition Analysis

The hydrogen bond in complexes involving Cl^-, F^-, and OH^- with HFIP was analyzed by the Morokuma-Ziegler energy decomposition analysis method (EDA).[21–24] Thereby the analysis included both the bond between the hydrogen atom of HFIP and the anions, as well as the bond between the hydrogen and oxygen atoms within HFIP. Furthermore, the hydrogen bonding in fluoride complexes with two HFIP molecules was also examined. EDA splits the system into fragments. The bonding energy is then decomposed by EDA into several physically meaningful contributions that enable a characterization of the chemical bond like the preparation energy (deformation of the fragments during the bond formation), dispersion contribution, quasiclassical electrostatic contribution, Pauli repulsion, and orbital contribution.

Structures were optimized and subjected to EDA calculation with the Amsterdam Modeling Suite (AMS, version 2024.105).[25] All DFT calculations were done with B3LYP and all electron TZP basis set.[26] Additionally, the Grimme D3-BJ dispersion correction[12,13] was used and scalar relativistic effects were treated by the zeroth order regular approximation (ZORA).[27] The numerical quality was set to "very good" which governs the density fitting and numerical integration. This numerical quality corresponds to 10^{-6} E_h as SCF convergence criterion. For the geometry optimization, the energy criterion accounted of $3\cdot10^{-3}$ E_h and the gradient criterion of 10^{-3} E_h $Å^{-1}$.

2. NaX Mass Spectra

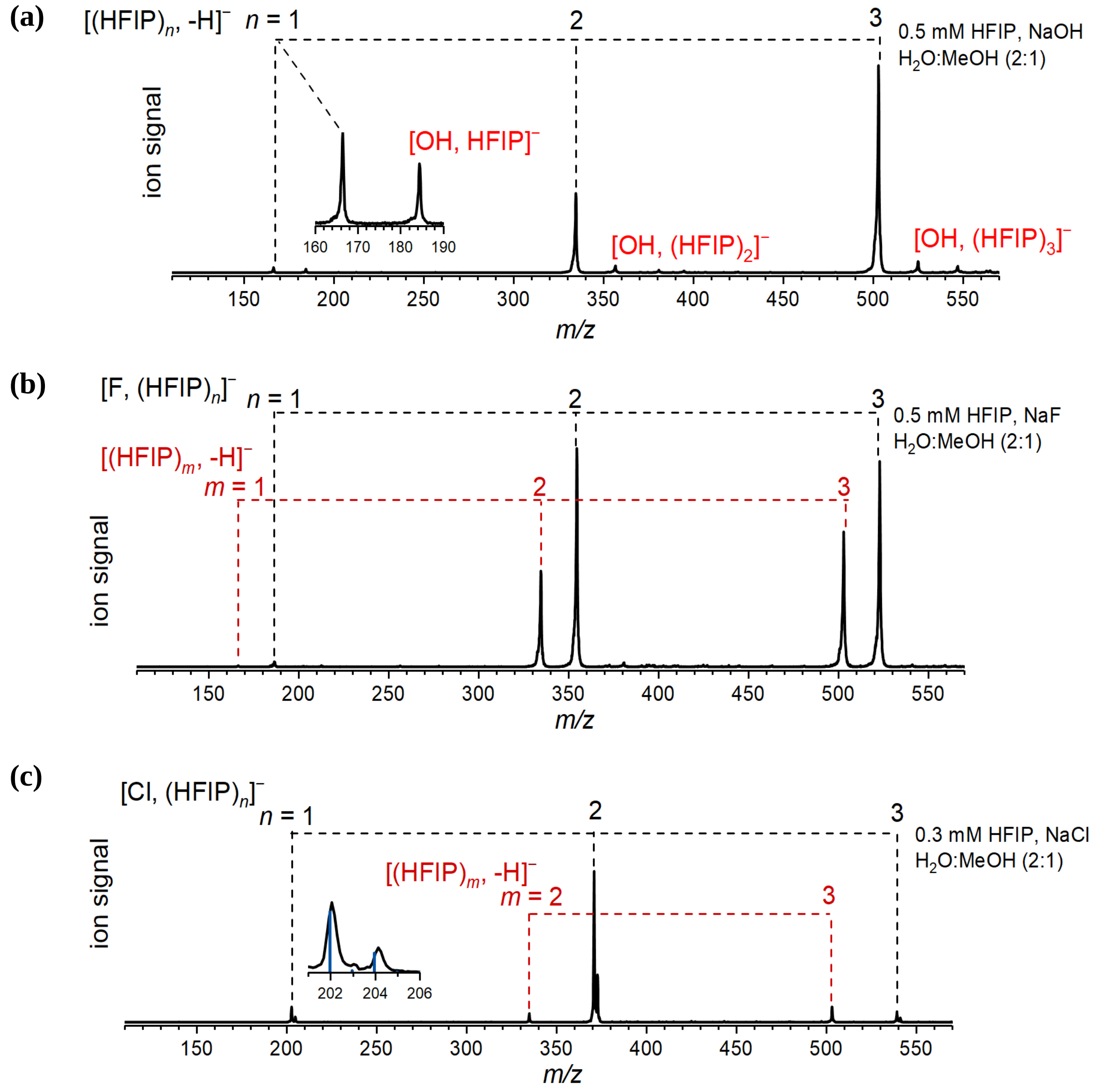

Figure S1. Quadrupole mass spectra from *m/z* 140 to *m/z* 560 obtained from NaX, HFIP solutions: a) NaOH; b) NaF; c) NaCl.

3. IRPD Spectra

3.1 Band Position

Table S2. IRPD band positions, harmonic B3LYP-D3(BJ)/def2-TZVPP (in cm^{-1}) and band assignments of the fundamental transitions.

$[HFIP_{-H}]^-$		$[HFIP_{-H}, H_2O]^-$		$[F, HFIP]^-$		$F^-(HFIP)_2$		$F^-(HFIP)_3$		$Cl^-(HFIP)$		$Cl^-(HFIP)_2$		Assign.
IRPD	B3LYP	IRPD	B3LYP	IRPD	B3LYP	IRPD	B3LYP[a]	IRPD	B3LYP[a]	IRPD	B3LYP	IRPD	B3LYP[a]	
2887	2589	2914	2744	2783	2852	2952	3030/3031	2862	3050/50/49	2936	3009	-	3044/42	ν_{CH}
1334	1324	1350	1354	1364	1359	1386	1393/86	1392	1400/1394/94	1395	1392	1393	1399/92	δ_{CCH}
1322	1359		1349	1307	1330		1364/61		1377/77/63	1324	1339		1342/1334	δ_{OCH}
1260	1233	1269	1253	1261	1259	1285	1280/77	1294	1287/83/83	1286	1277	1288	1282/79	ν_{CC}
1241	1212	1249	1231		1235	1268	1254/53	1271	1257/57/57	1264	1248	1169	1255/55	
1229	1271	1207	1234	1155	1205	1163	1179(s)<br>1175(a)	1155	1159 (s)<br>1156/56 (a)	1162	1165	1157	1159 (s)<br>1155 (a)	ν_{CO}[b]
1185	1156	1195	1171	1209	1224	1226	1201/00	1229	1206/03/03	1233	1209	1230	1208/04	ν_{CF}
1133	1097	1143	1118	1131	1163	1183	1161/61	1194	1176/76/68	1173	1149	1184	1167/65	
1089	1057	1099	1070	1089	1125	1144	1119/18	1141	1129/29/29	1144	1115	1139	1126/24	
1068	1032	1079	1050	1077	1099	1104	1087/82	1108	1095/87/87	1102	1078	1106	1090/85	
		2632 (HB)<br>3699(Free)	2936<br>3866	1436[d]	1951	1790,<br>2102	2706 (s)<br>2361(a)	2601	3185 (s)<br>2930/30 (a)	2535	2840	2991	3208 (s)<br>3127 (a)	ν_{OH}[b,c]
		1654	1698	1543	1411	1527	1599/80	1512	1564/64/44	1453	1509		1496/94	δ_{COH}

[a] Due to symmetry some modes are predicted just a few (1 to 6 cm^{-1}) wavenumbers a part. All the calculated frequencies are reported here with XXXX/YY(/ZZ) representing the two (or three) values, XXXX, XXYY (and XXZZ).

[b] For the symmetric structures, (s) represents totally symmetric modes and (a) antisymmetric modes.

[c] For the $[HFIP_{-H}, H_2O]^-$ complex the two ν_{OH} reported are from the water molecule. They are identified as HB (hydrogen-bonded) and Free.

[d] Reported value is the average of the two peaks observed at 1429 cm^{-1} and 1443 cm^{-1} as reported in Table S3

3.2 MP2 vs B3LYP

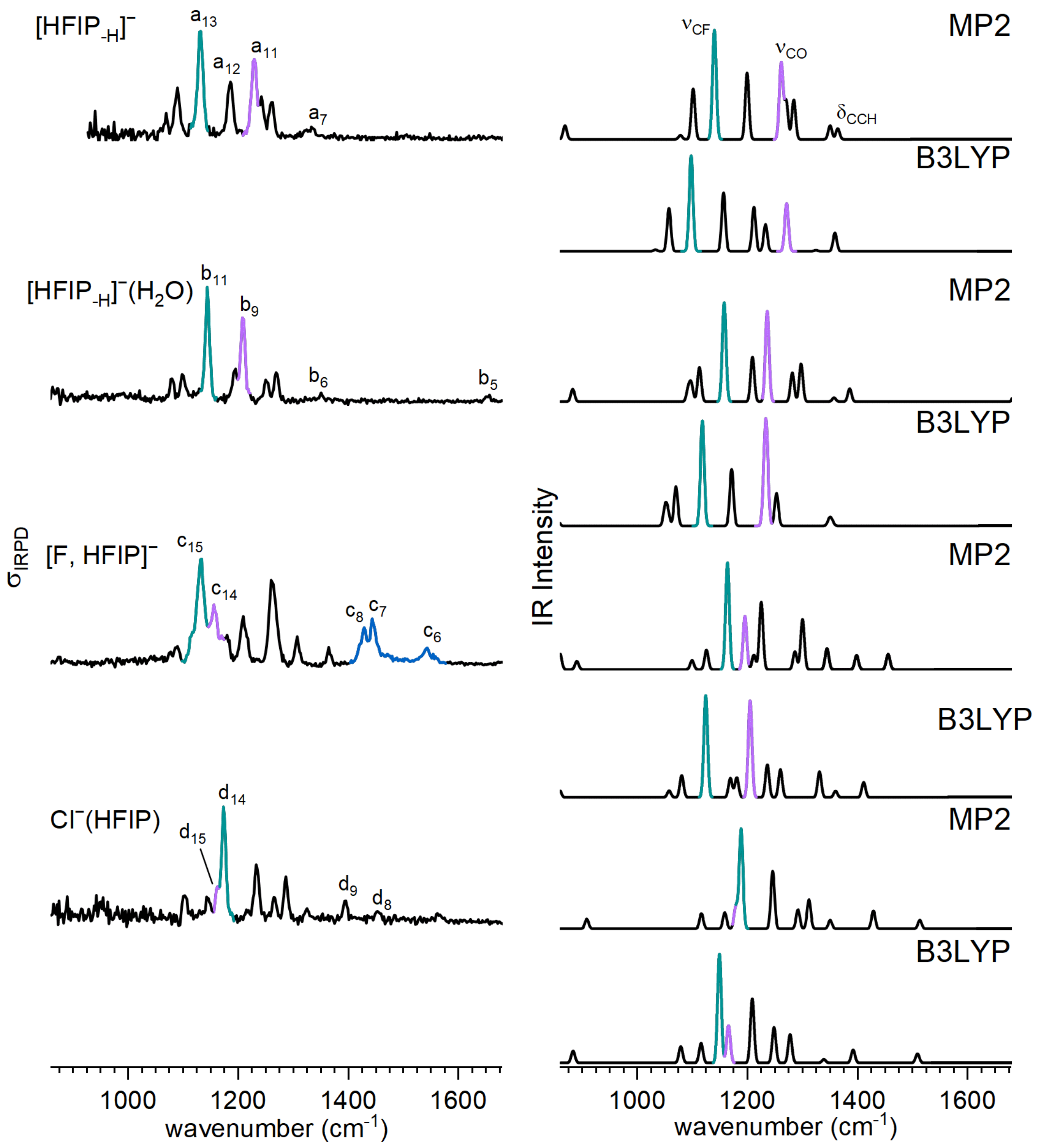

Figure S2. - MP2/aug-cc-pVTZ (top) and B3LYP-D3(BJ)/def2-TZVPP (bottom) spectra of $[HFIP_{-H}]^-$, $[HFIP_{-H}]^-(H_2O)$, $[F, HFIP]^-$, and $Cl^-(HFIP)$ in the spectral range of 850 cm^{-1} to 1800 cm^{-1} compared to respective IRPD spectra of tagged complexes. Colored the prognostic bands assigned: the most IR active CF stretch (ν_{CF}, green), the CO stretch (ν_{CO} , lilac) and the OH stretch (blue). See Table S2 for complete assignment.

3.3 Extended Spectral Region (2250 cm^{-1} to 4000 cm^{-1})

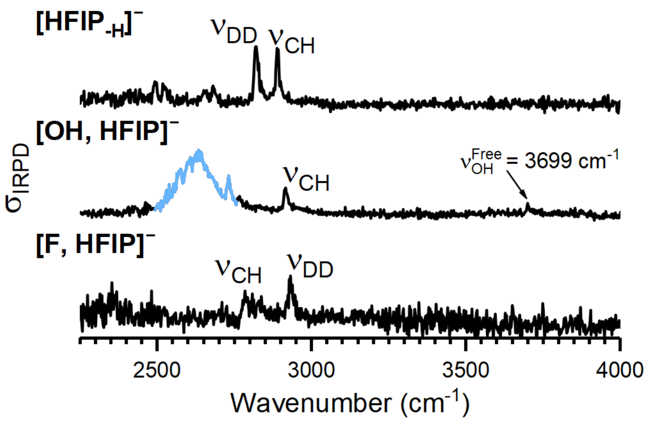

Figure S3. IRPD spectra of D_2-tagged $[HFIP_{-H}]^-$, $[OH, HFIP]^-$, and $[F, HFIP]^-$ in the spectral range from 2250 cm^{-1} to 4000 cm^{-1} recorded at 13 K. Highlighted in blue is the hydrogen-bonded OH stretching band of the water molecule (see Table S3 for band assignments and Table S5 for structure parameters).

Table S3. - Band labels IRPD band positions (in cm^{-1}), harmonic MP2/aug-cc-pVTZ and B3LYP/D3(BJ)/def2-TZVPP vibrational frequencies (in cm^{-1}) and band assignments of the fundamental transitions.

**$[HFIP_{-H}]^-$**				**$[HFIP, OH]^-$**				**$[F, HFIP]^-$**				**$[Cl, HFIP]^-$**				
**Label**	IRPD	MP2.	B3LYP	Label	IRPD	MP2	B3LYP	Label	IRPD	MP2	B3LYP	Label	IRPD	MP2	B3LYP	
a_1	2887	2733	2589	b_2	2914	2874	2744	c_1	2783	2948	2852	d_1	2936	2926	3009	ν_{CH}
a_7	1334	1364	1324	b_6	1350	1385	1354	c_9	1364	1398	1359	d_9	1395	1429	1392	δ_{CCH}
$a_{8,}$	1322	1350	1359	-	-	1357	1349	c_{10}	1307	1344	1330	d_{10}	1324	1350	1339	δ_{OCH}
$a_{9,}$	1260	1284	1233	b_7	1269	1297	1253	c_{11}	1261	1300	1259	d_{11}	1286	1311	1277	ν_{CC}
a_{10}	1241	1270	1212	b_8	1249	1281	1231			1286	1235	d_{12}	1264	1291	1248	
a_{11}	1229	1261	1270	b_9	1207	1236	1234	c_{14}	1155	1195	1205	d_{15}	1162	1179	1165	ν_{CO}
a_{12}	1185	1199	1156	b_{10}	1195	1209	1171	c_{12}	1209	1224	1180	d_{13}	1233	1246	1209	
a_{13}	1132	1140	1097	b_{11}	1143	1157	1118	c_{15}	1131	1163	1124	d_{14}	1173	1188	1149	
a_{14}	1089	1101	1057	b_{12}	1099	1112	1070	c_{16}	1089	1125	1080	d_{16}	1144	1159	1115	ν_{CF}
a_{15}	1068	1078	1032	b_{13}	1079	1092	1050	c_{17}	1077	1099	1057	d_{17}	1102	1116	1078	
				b_4	2632	2803	2936	c_8, c_7	1443-1429	1738	1951	d_3	2535	2925	2840	ν_{OH}
				-	-	-		c_6	1543	1455	1411	d_8	1453	1513	1509	δ_{COH}
				b_1	3699	3891	3866									ν_{OH}^{Free}
				b_5	1654	1687	1698									δ_{HOH}

4. Geometrical Parameters

Table S4. Selected B3LYP-D3(BJ)/def2-TZVPP geometrical parameters: OH distance d_{OH}, HB distance d_{HX} (both in pm), HB angle θ_{OHX} and dihedral angle φ_{HCOH} (both in degrees) for [X, (HFIP)$_n$]$^-$

	**[HFIP$_{-\mathrm{H}}$]$^-$**	**[OH, HFIP]$^-$**	**[F, HFIP]$^-$**	**[F, (HFIP)$_2$]$^-$**	**[F, (HFIP)$_3$]$^-$**	**[Cl, HFIP]$^-$**	**[Cl, (HFIP)$_2$]$^-$**
d_{OH}		161	134	103	100	102	100
d_{HX}		101	105	140	151	194	200
d_{OX}		262	238	243	250	293	298
θOHX		173	176	173	171	163	166
rCO	130	132	134	137	138	138	138
φHCOH		20.6	0	1.5	12	0	0

Table S5. - Selected B3LYP-D3(BJ)/def2-TZVPP and MP2/aug-cc-pVTZ geometrical parameters: OH distance d_{OH}, HB distance d_{HX} (both in pm), HB angle θ_{OHX} and dihedral angle φ_{HCOH} (both in degrees) for [X, (HFIP)$_n$]$^-$

	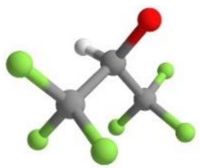		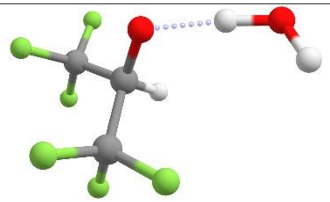		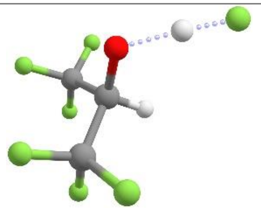		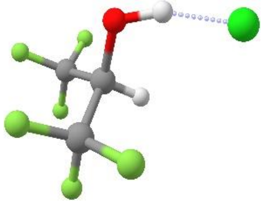	
	[HFIP$_{-\mathrm{H}}$]$^-$		[OH, HFIP]$^-$		[F, HFIP]$^-$		[Cl, HFIP]$^-$	
Method	B3LYP	MP2	B3LYP	MP2	B3LYP	MP2	B3LYP	MP2
d_{OH}			161	157	134	130	102	101
d_{HX}			101	101	105	106	194	192
d_{OX}			262	259	238	236	293	290
θOHX			173	174	176	176	163	161
rCO	130	132	132	133	134	135	138	138
φHCOH			20.6	16	0	0	0	0

5. Higher Energy Isomers

5.1 [F, (HFIP)$_2$]$^-$

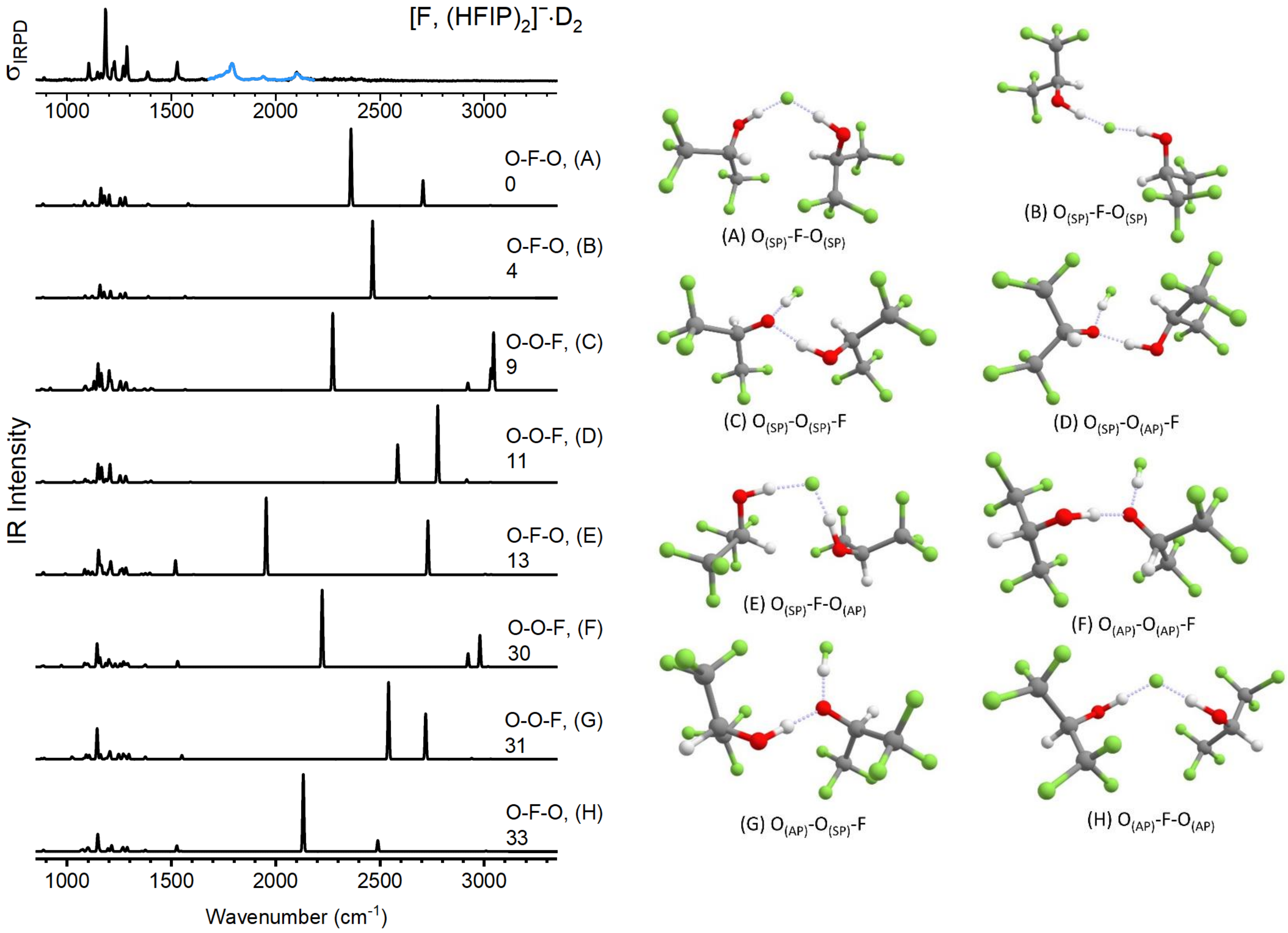

Figure S4. – (left) IRPD spectrum of D_2-tagged [F, (HFIP)$_2$]$^-$ in the spectral range of 950 cm^{-1} to 3350 cm^{-1} recorded at 13 K (top) compared to unscaled, harmonic B3LYP-D3(BJ)/def2-TZVPP IR spectra of the corresponding untagged complexes (A-H), indicating the H-bonding motif and relative energy given in kJ mol^{-1}. (right) Geometry of complexes A-H indicating HFIP geometry relative to the HCOH dihedral angle, antiperiplanar (AP) and synperiplanar (SP), as well as H-bonding motif. xyz coordinates of optimized geometries are shared in Zenodo.

5.2 [F, (HFIP)$_3$]$^-$

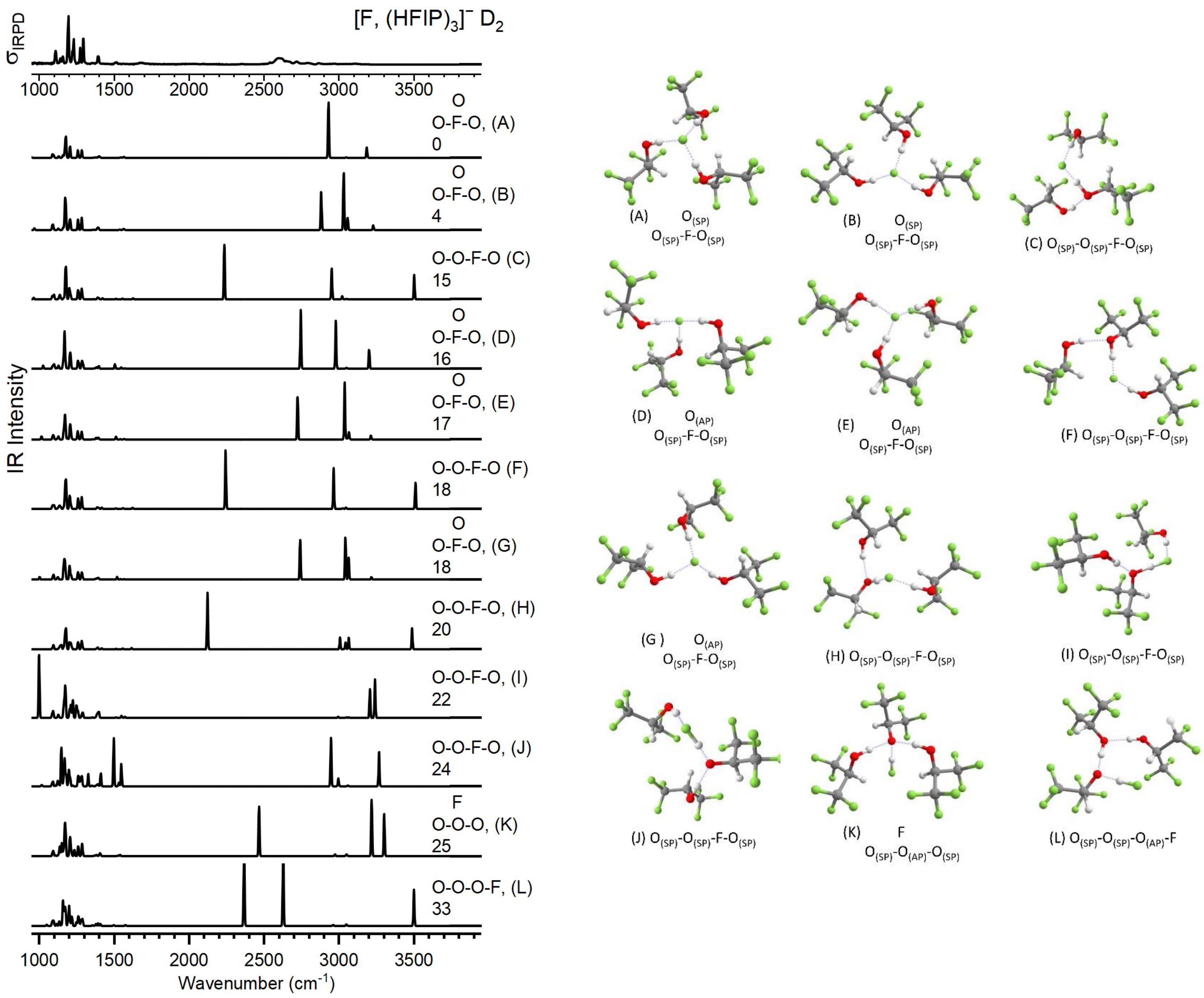

Figure S5. – (left) IRPD spectrum of D_2-tagged [F, (HFIP)$_3$]$^-$ in the spectral range of 950 cm^{-1} to 3350 cm^{-1} recorded at 13 K (top) compared to unscaled, harmonic B3LYP-D3(BJ)/def2-TZVPP IR spectra of the corresponding untagged complexes (A-L), indicating the H-bonding motif and relative energy given in kJ mol^{-1}. (right) Geometry of complexes A-L indicating HFIP geometry relative to the HCOH dihedral angle, antiperiplanar (AP) and synperiplanar (SP), as well as H-bonding motif. xyz coordinates of optimized geometries are shared in Zenodo.

5.3 [Cl, $(HFIP)_2$]⁻

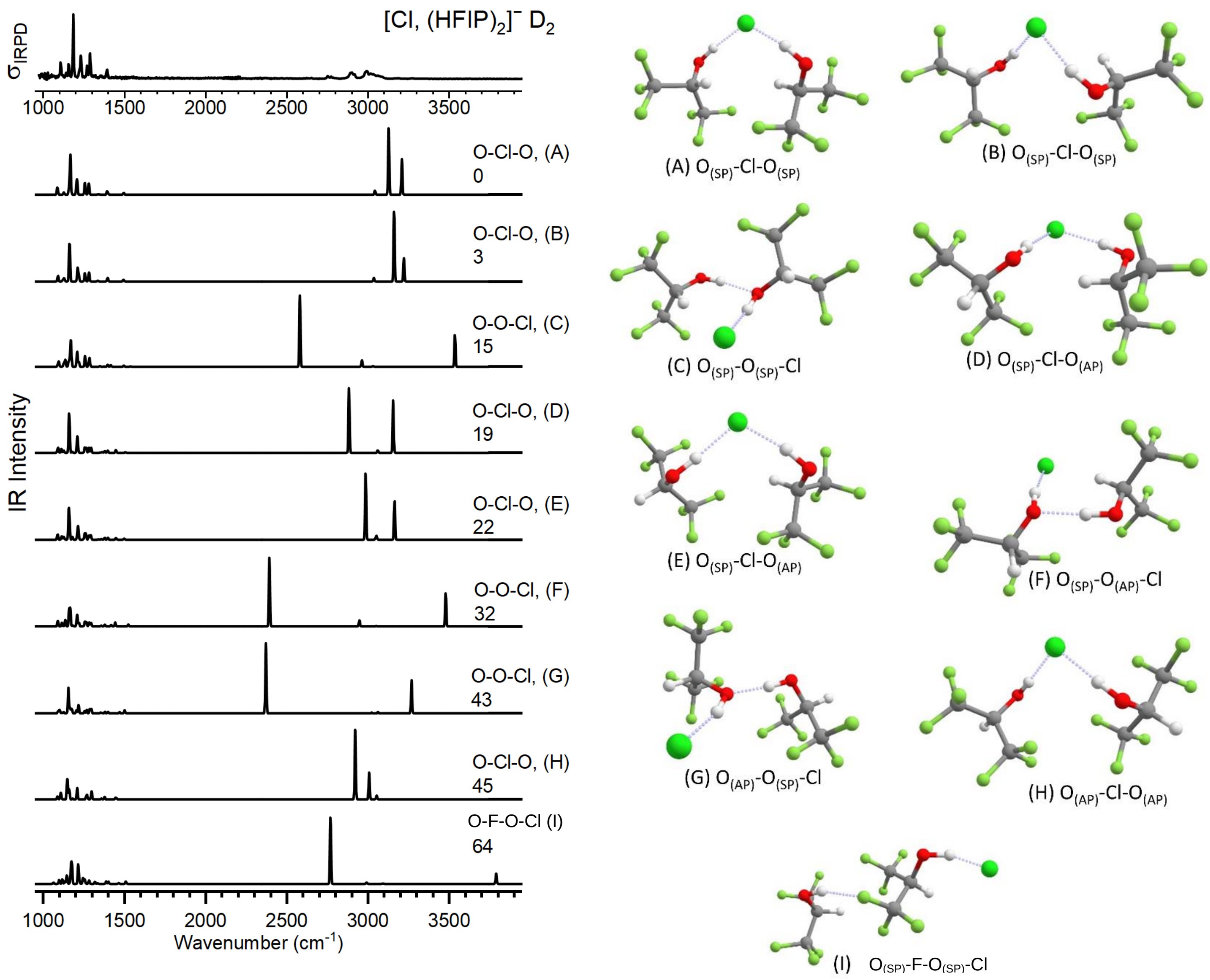

Figure S6. – (left) IRPD spectrum of D_2-tagged [Cl, $(HFIP)_2$]⁻ in the spectral range of 950 cm^{-1} to 3350 cm^{-1} recorded at 13 K (top) compared to unscaled, harmonic B3LYP-D3(BJ)/def2-TZVPP IR spectra of the corresponding untagged complexes (A-H), indicating the H-bonding motif and relative energy given in kJ mol^{-1}. (right) Geometry of complexes A-H indicating HFIP geometry relative to the HCOH dihedral angle, antiperiplanar (AP) and synperiplanar (SP) as well as H-bonding motif. xyz coordinates of optimized geometries are shared in Zenodo.

6. Expectation Values for the OH Position for Vibrational Levels 0 to 5

The expectation value for OH position is determined as

$$\langle d_i^{OH} \rangle = \left(\int_{-0.8}^{b} \Psi_i^* x^{OH} \Psi_i dx + x_{min} \right) / a_0$$

where Ψ_i is the wavefunction for the ith-vibrational level, a_0 is Bohr radius. x_{min} (to correct for the potential shift) and the upper integration limit are given in Table S6.

Table S6 – Equilibrium heavy atom distance (d_e^{OX}, all distances in pm), equilibrium RO-H distance d_e^{OH}, and expectation values of the RO-H distance in the vibrational ground state $\langle d_0^{OH} \rangle$ as well as the first five vibrational excited states ($\langle d_{1-5}^{OH} \rangle$).

	**Cl⁻**	**F⁻**	**OH⁻**
x_{min}	1.915	2.200	2.200
b	1.8	1.2	1.3
d_e^{OX}	290	236	266
d_e^{OH}	101	130	165
$\langle d_0^{OH} \rangle$	104	126	156
$\langle d_1^{OH} \rangle$	112	121	146
$\langle d_2^{OH} \rangle$	120	121	133
$\langle d_3^{OH} \rangle$	131	120	130
$\langle d_4^{OH} \rangle$	137	120	130
$\langle d_5^{OH} \rangle$	139	120	130

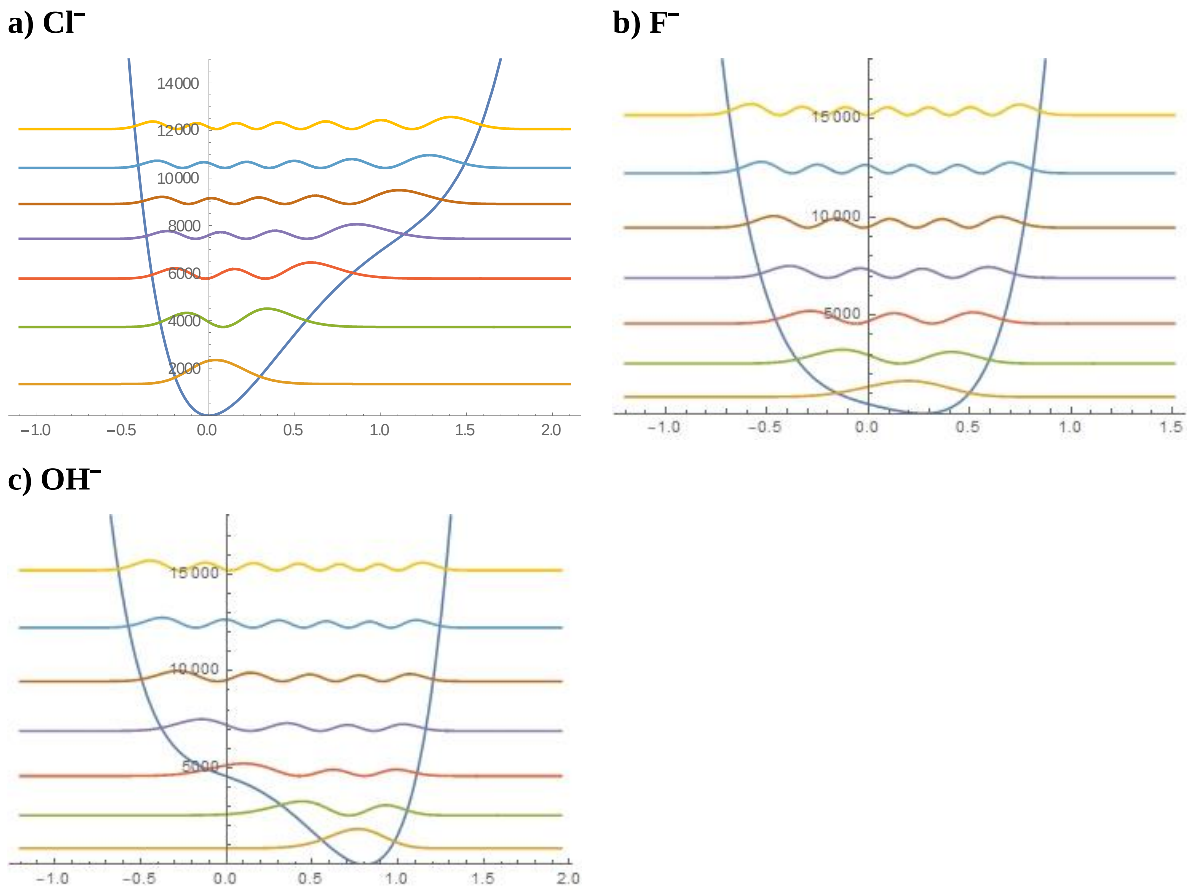

Figure S7. – Fitted potential and Ψ^2, shifted x-axis in atomic units (Bohr) and y-axis represented in cm^{-1}. The estimated dissociation energy D_e (without counterpoise and basis set superposition error corrections) are Cl^- 135 kJ mol^{-1} (11 315 cm^{-1}), F^-125 kJ mol^{-1} (10 487 cm^{-1}) and OH^- 72 kJ mol^{-1} (5979 cm^{-1}). For Cl- and OH- the estimated D_es coincide with the vibrational level where the degree of proton transfer and proton delocalization would increase in the above unrelaxed 1D-potentials.

7. [F, *i*-PrOH]⁻ complex

7.1 IRPD spectrum of [F, *i*-PrOH]⁻ complex

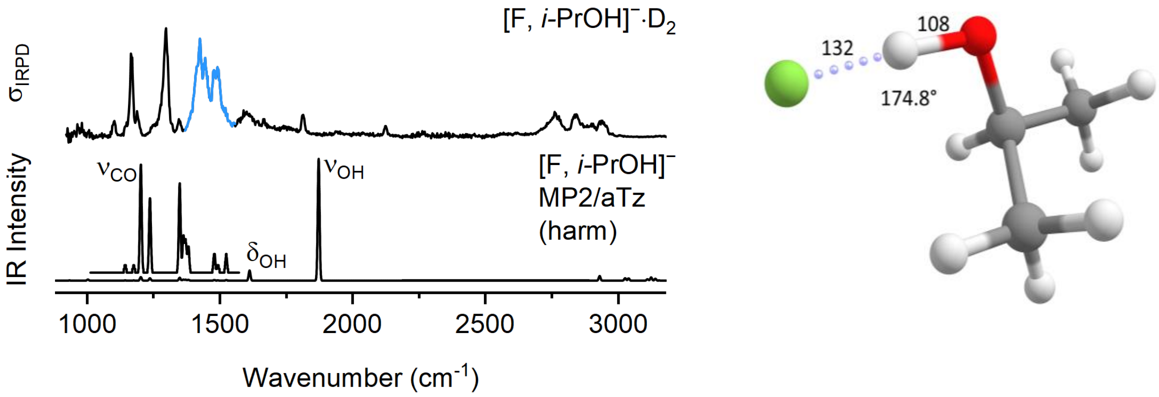

Figure S8. – IRPD spectrum of D_2-tagged [F, *i*-PrOH]⁻ in the spectral range of 950 cm^{-1} to 3200 cm^{-1} recorded at 13 K (top) compared to unscaled, harmonic MP2/aug-cc-pVTZ spectrum of the corresponding untagged complex. The bands highlighted in blue have been preliminarily assigned to the OH stretching and OH bending fundamentals. Note that strong coupling with the CH bending modes is expected in this range. The presence of a second isomer (not shown) cannot be discarded as for other X⁻(*i*-PrOH) complexes previously reported.[28]

7.2 Energy Decomposition Analysis for [F, *i*-PrOH]$^-$

Table S7. EDA results for two possible fragmentation channels of the [F, *i*-PrOH]$^-$ complexes. Fragmentation leads to the formation of either deprotonated *i*-PrO$^-$ + HF or intact *i*-PrOH + F$^-$, labelled D and I, respectively. Energies are given in kJ mol^{-1} and bond distance in pm.

	Isopropanol			
	**I**		**D**	
ΔE_{int}	-729		-188	
ΔE_{int}(disp)^b	-1	2%	-5	5%
ΔE_{int}(elec)^b	-728	98%	-183	95%
ΔE_{Pauli}	510		217	
$\Delta E_{elstat}{}^c$	-142	36%	-213	64%
$\Delta E_{orb}{}^c$	-1097	64%	-187	36%
ΔE_{prep}	541		27	
E_{bond}	-188		-161	
**d(X-H)**	107		136	

8. Table $\Delta\nu_{OH}$ and ΔPA

Table S8 – Proton affinities from Ref [29] (in kJ mol^{-1}) and OH shifts (cm^{-1}). For the solvents we report the PA of the conjugated base, which equals the enthalpy of deprotonation for each solvent.

	**PA**	**H_2O (1633)**		**HFIP (1443)**		***i*-PrOH (1569)**	
		ΔPA	$\Delta\nu_{OH}$	ΔPA	$\Delta\nu_{OH}$	ΔPA	$\Delta\nu_{OH}$
**I$^-$**	1315	-307	290	-128	640	-254	343
**Br$^-$**	1353	-269	399	-90	920	-216	450
**Cl$^-$**	1395	-227	549	-48	1133	-174	571
**F$^-$**	1555	-108	2211	112	2232	-14	2256
**(HFIP$_{-H}$)$^-$**	1443	-190	1075				
**OH$^-$**	1633	0	3010				

[a] Red-shift $\Delta\nu_{OH}$ = Free ν_{OH} – Bonded ν_{OH}, where Free ν_{OH} are 3668 cm^{-1} (HFIP)[30], 3707 cm^{-1} (H_2O)[31] and 3658 cm^{-1} (*i*-PrOH)[32].

9. References

(1) Heine, N.; Asmis, K. R. Cryogenic ion trap vibrational spectroscopy of hydrogen-bonded clusters relevant to atmospheric chemistry. *Int. Rev. Phys. Chem.* **2015**, *34* (1), 1–34. DOI: 10.1080/0144235X.2014.979659.

(2) Brümmer, M.; Kaposta, C.; Santambrogio, G.; Asmis, K. R. Formation and photodepletion of cluster ion–messenger atom complexes in a cold ion trap: Infrared spectroscopy of VO+, VO2+, and VO3+. *J. Chem. Phys* **2003**, *119* (24), 12700–12703. DOI: 10.1063/1.1634254.

(3) Mayer, M.; Asmis, K. R. Online Monitoring of Isomeric Reaction Intermediates. *J. Phys. Chem. A* **2021**, *125* (14), 2801–2815. DOI: 10.1021/ACS.JPCA.0C11371.

(4) Bosenberg, W. R.; Guyer, D. R. Broadly tunable, single-frequency optical parametric frequency-conversion system. *J. Opt. Soc. Am. B* **1993**, *10* (9), 1716. DOI: 10.1364/JOSAB.10.001716.

(5) Heine, N.; Asmis, K. R. Cryogenic Ion Trap Vibrational Spectroscopy of Hydrogen-Bonded Clusters Relevant to Atmospheric Chemistry (International Reviews in Physical Chemistry , 2015, Vol. 34, No. 1, 1–34). *Int. Rev. Phys. Chem.* **2016**, *35* (3), 507. DOI: 10.1080/0144235X.2016.1203533.

(6) Neese, F. The ORCA program system. *WIREs. Comput. Mol. Sci.* **2012**, *2* (1), 73–78. DOI: 10.1002/wcms.81.

(7) Neese, F. Software update: The ORCA program system—Version 5.0. *WIREs. Comput. Mol. Sci.* **2022**, *12* (5). DOI: 10.1002/wcms.1606.

(8) Pracht, P.; Bohle, F.; Grimme, S. Automated exploration of the low-energy chemical space with fast quantum chemical methods. *Phys. Chem. Chem. Phys.* **2020**, *22* (14), 7169–7192. DOI: 10.1039/C9CP06869D.

(9) Bannwarth, C.; Ehlert, S.; Grimme, S. GFN2-xTB-An Accurate and Broadly Parametrized Self-Consistent Tight-Binding Quantum Chemical Method with Multipole Electrostatics and Density-Dependent Dispersion Contributions. *J. Chem. Theory Comput.* **2019**, *15* (3), 1652–1671. DOI: 10.1021/acs.jctc.8b01176. Published Online: Feb. 11, 2019.

(10) Frisch, M. J.; Trucks, G. W.; Schlegel, H. B.; Scuseria, G. E.; Robb, M. A.; Cheeseman, J. R.; Scalmani, G.; Barone, V.; Petersson, G. A.; Nakatsuji, H.; Li, X.; Caricato, M.; Marenich, A. V.; Bloino, J.; Janesko, B. G.; Gomperts, R.; Mennucci, B.; Hratchian, H. P.; Ortiz, J. V.; Izmaylov, A. F.; Sonnenberg, J. L.; Williams; Ding, F.; Lipparini, F.; Egidi, F.; Goings, J.; Peng, B.; Petrone, A.; Henderson, T.; Ranasinghe, D.; Zakrzewski, V. G.; Gao, J.;

Rega, N.; Zheng, G.; Liang, W.; Hada, M.; Ehara, M.; Toyota, K.; Fukuda, R.; Hasegawa, J.; Ishida, M.; Nakajima, T.; Honda, Y.; Kitao, O.; Nakai, H.; Vreven, T.; Throssell, K.; Montgomery Jr., J. A.; Peralta, J. E.; Ogliaro, F.; Bearpark, M. J.; Heyd, J. J.; Brothers, E. N.; Kudin, K. N.; Staroverov, V. N.; Keith, T. A.; Kobayashi, R.; Normand, J.; Raghavachari, K.; Rendell, A. P.; Burant, J. C.; Iyengar, S. S.; Tomasi, J.; Cossi, M.; Millam, J. M.; Klene, M.; Adamo, C.; Cammi, R.; Ochterski, J. W.; Martin, R. L.; Morokuma, K.; Farkas, O.; Foresman, J. B.; Fox, D. J. *Gaussian 16 Rev. C.01*, 2016.

(11) Becke, A. D. Density-functional thermochemistry. III. The role of exact exchange. *J. Chem. Phys* **1993**, *98* (7), 5648–5652. DOI: 10.1063/1.464913.

(12) Grimme, S.; Antony, J.; Ehrlich, S.; Krieg, H. A consistent and accurate ab initio parametrization of density functional dispersion correction (DFT-D) for the 94 elements H-Pu. *J. Chem. Phys* **2010**, *132* (15). DOI: 10.1063/1.3382344.

(13) Grimme, S.; Ehrlich, S.; Goerigk, L. Effect of the damping function in dispersion corrected density functional theory. *J. Comput. Chem.* **2011**, *32* (7), 1456–1465. DOI: 10.1002/jcc.21759.

(14) Weigend, F.; Ahlrichs, R. Balanced basis sets of split valence, triple zeta valence and quadruple zeta valence quality for H to Rn: Design and assessment of accuracy. *Phys. Chem. Chem. Phys.* **2005**, *7* (18), 3297–3305. DOI: 10.1039/b508541a.

(15) Frisch, M. J.; Head-Gordon, M.; Pople, J. A. A direct MP2 gradient method. *Chem. Phys. Lett.* **1990**, *166* (3), 275–280. DOI: 10.1016/0009-2614(90)80029-D.

(16) Frisch, M. J.; Head-Gordon, M.; Pople, J. A. Semi-direct algorithms for the MP2 energy and gradient. *Chem. Phys. Lett.* **1990**, *166* (3), 281–289. DOI: 10.1016/0009-2614(90)80030-H.

(17) Head-Gordon, M.; Head-Gordon, T. Analytic MP2 frequencies without fifth-order storage. Theory and application to bifurcated hydrogen bonds in the water hexamer. *Chem. Phys. Lett.* **1994**, *220* (1-2), 122–128. DOI: 10.1016/0009-2614(94)00116-2.

(18) Head-Gordon, M.; Pople, J. A.; Frisch, M. J. MP2 energy evaluation by direct methods. *Chem. Phys. Lett.* **1988**, *153* (6), 503–506. DOI: 10.1016/0009-2614(88)85250-3.

(19) Dunning, T. H. Gaussian basis sets for use in correlated molecular calculations. I. The atoms boron through neon and hydrogen. *J. Chem. Phys* **1989**, *90* (2), 1007–1023. DOI: 10.1063/1.456153.

(20) Woon, D. E.; Dunning, T. H. Gaussian basis sets for use in correlated molecular calculations. III. The atoms aluminum through argon. *J. Chem. Phys* **1993**, *98* (2), 1358–1371. DOI: 10.1063/1.464303.

(21) Bickelhaupt, F. M.; Baerends, E. J. Kohn-Sham Density Functional Theory: Predicting and Understanding Chemistry. In *Reviews in Computational Chemistry*; Lipkowitz, K. B., Boyd, D. B., Eds.; Reviews in Computational Chemistry; Wiley, 2000; pp 1–86. DOI: 10.1002/9780470125922.ch1.

(22) Ziegler, T.; Rauk, A. On the calculation of bonding energies by the Hartree Fock Slater method. *Theor. Chem. Acc.* **1977**, *46* (1), 1–10. DOI: 10.1007/BF02401406.

(23) Kitaura, K.; Morokuma, K. A new energy decomposition scheme for molecular interactions within the Hartree-Fock approximation. *Int. J. Quantum Chem.* **1976**, *10* (2), 325–340. DOI: 10.1002/qua.560100211.

(24) Zhao, L.; Hopffgarten, M. von; Andrada, D. M.; Frenking, G. Energy decomposition analysis. *WIREs. Comput. Mol. Sci.* **2018**, *8* (3). DOI: 10.1002/wcms.1345.

(25) te Velde, G.; Bickelhaupt, F. M.; Baerends, E. J.; Fonseca Guerra, C.; van Gisbergen, S. J. A.; Snijders, J. G.; Ziegler, T. Chemistry with ADF. *J. Comput. Chem.* **2001**, *22* (9), 931–967. DOI: 10.1002/jcc.1056.

(26) van Lenthe, E.; Baerends, E. J. Optimized Slater-type basis sets for the elements 1-118. *J. Comput. Chem.* **2003**, *24* (9), 1142–1156. DOI: 10.1002/jcc.10255.

(27) van Lenthe, E.; Ehlers, A.; Baerends, E.-J. Geometry optimizations in the zero order regular approximation for relativistic effects. *J. Chem. Phys* **1999**, *110* (18), 8943–8953. DOI: 10.1063/1.478813.

(28) Barp, M.; Kreuter, F.; Huang, Q.-R.; Jin, J.; Ninov, F. E.; Kuo, J.-L.; Tonner-Zech, R.; Asmis, K. R. Quantifying Hexafluoroisopropanol's Hydrogen Bond Donor Ability: Infrared Photodissociation Spectroscopy of Halide Anion HFIP Complexes. *Chem. Sci.* **2025** (16), 5174–5185. DOI: 10.1039/D4SC08456J.

(29) P.J. Linstrom and W.G. Mallard, Ed. *"Proton Affinity Evaluation" in NIST Chemistry WebBook, NIST Standard Reference Database Number 69*.

(30) Barnes, A. J.; Murto, J. Infra-red cryogenic studies. Part 10.—Conformational isomerism of 1,1,1,3,3,3-hexafluoropropan-2-ol. *J. Chem. Soc., Faraday Trans. 2* **1972**, *68* (0), 1642–1651. DOI: 10.1039/F29726801642.

(31) Gordon, I. E.; Rothman, L. S.; Hargreaves, R. J.; Hashemi, R.; Karlovets, E. V.; Skinner, F. M.; Conway, E. K.; Hill, C.; Kochanov, R. V.; Tan, Y.; Wcisło, P.; Finenko, A. A.; Nelson,

K.; Bernath, P. F.; Birk, M.; Boudon, V.; Campargue, A.; Chance, K. V.; Coustenis, A.; Drouin, B. J.; Flaud, J.-M.; Gamache, R. R.; Hodges, J. T.; Jacquemart, D.; Mlawer, E. J.; Nikitin, A. V.; Perevalov, V. I.; Rotger, M.; Tennyson, J.; Toon, G. C.; Tran, H.; Tyuterev, V. G.; Adkins, E. M.; Baker, A.; Barbe, A.; Canè, E.; Császár, A. G.; Dudaryonok, A.; Egorov, O.; Fleisher, A. J.; Fleurbaey, H.; Foltynowicz, A.; Furtenbacher, T.; Harrison, J. J.; Hartmann, J.-M.; Horneman, V.-M.; Huang, X.; Karman, T.; Karns, J.; Kassi, S.; Kleiner, I.; Kofman, V.; Kwabia–Tchana, F.; Lavrentieva, N. N.; Lee, T. J.; Long, D. A.; Lukashevskaya, A. A.; Lyulin, O. M.; Makhnev, V.; Matt, W.; Massie, S. T.; Melosso, M.; Mikhailenko, S. N.; Mondelain, D.; Müller, H.; Naumenko, O. V.; Perrin, A.; Polyansky, O. L.; Raddaoui, E.; Raston, P. L.; Reed, Z. D.; Rey, M.; Richard, C.; Tóbiás, R.; Sadiek, I.; Schwenke, D. W.; Starikova, E.; Sung, K.; Tamassia, F.; Tashkun, S. A.; Vander Auwera, J.; Vasilenko, I. A.; Vigasin, A. A.; Villanueva, G. L.; Vispoel, B.; Wagner, G.; Yachmenev, A.; Yurchenko, S. N. The HITRAN2020 molecular spectroscopic database. *J. Quant. Spectrosc. Radiat. Transfer* **2022**, *277*, 107949. DOI: 10.1016/j.jqsrt.2021.107949.

(32) Schaal, H.; Häber, T.; Suhm, M. A. Hydrogen Bonding in 2-Propanol. The Effect of Fluorination. *J. Phys. Chem. A* **2000**, *104* (2), 265–274. DOI: 10.1021/jp9928558.
