# Supplementary material for: Infrared Photodissociation Spectroscopy of Fluoride–Anion Hexafluoroisopropanol Complexes: Solvation-Suppressed Proton Transfer
Source: J Phys Chem Lett. 2025 Jul 7;16(28):7187–93. doi: 10.1021/acs.jpclett.5c00953 (PMC12278305; doi:10.1021/acs.jpclett.5c00953)
Supplement: Supplementary file 2 [file jz5c00953_si_002.pdf]

jz-2025-00953a.R1

Name: Peer Review Information for "Infrared Photodissociation Spectroscopy of Fluoride Anion Hexafluoroisopropanol Complexes: Solvation-suppressed Proton Transfer"

First Round of Reviewer Comments

Reviewer: 1

Comments to the Author

The manuscript reported the characteristic hydrogen bonds in the complexes of fluoride anion with hexafluoroisopropanol (HFIP) using IR photodissociation spectroscopy with the D₂ tagging technique, which is combined with the quantum chemical computation. The size dependence of HFIP on the hydrogen bonding nature, including the proton transfer, was explored by observing the OH stretching redshift. A combination of experiment and computation revealed that suppression of the proton transfer to the fluorine anion with increasing HFIP ligands comes from the concomitant and symmetric H-bonds due to the strong donor ability of HFIP. Furthermore, the computation of both an energy decomposition analysis and a potential energy scan provided useful information about the intermolecular interaction between fluoride anion and HFIP.

1. What is the major advance reported in the paper?

The major advance in this study is the elucidation of the size dependence of ionic hydrogen bonds on fluoride anion complex with HFIP. This study of micro-solvated systems will provide a new insight into fluorination chemistry.

2. What is the immediate significance of this advance?

Comparative measurement of [F, HFIP]⁻ with [HFIP -H]⁻ and [OH, HFIP]⁻ successfully revealed the extraordinary redshifted OH stretch due to the proton transfer. The vibrational signatures at 1100 – 1400 cm⁻¹ consist of the intramolecular backbone normal modes of the deprotonated HFIP moiety. This

vibrational signature is completely different from that of [Cl, HFIP]⁻ which exhibits the neutral HFIP. The OH stretch at 1450 cm⁻¹ was probably difficult to identify without the comparative measurement because of the complicated band signature in this region. Precise experiment and computation successfully revealed the proton transfer in [F, HFIP]⁻ and its suppression with increasing the number of HFIP.

In conclusion, this manuscript is well-written, with reliable experimental and theoretical data. I recommend this study be published in The Journal of Physical Chemistry Letters with a minor revision. It would be better if the authors addressed the comments below.

3. Technical suggestions

The authors presented the DFT minimum-energy structures of [F, (HFIP)₁₋₃]⁻ in Figure 2 and ESI. I understand that the ionic hydrogen bonds are caused by the strong donor ability of the HFIP molecule. This ability is ascribed to the fluorination of the methyl groups in iso-propanol (i-PrOH). Thus, if the structures and interaction energies of [F, (HFIP)₁₋₃]⁻ are compared to those of [F, (i-PrOH)₁₋₃]⁻, we can probably understand the donor ability of HFIP more quantitatively. The author already reported the comparative study of HFIP and i-PrOH for the other halide anion complexes in reference 22. If the authors feel that such consideration is necessary, I would encourage them to revise it.

Reviewer: 2

Comments to the Author

The manuscript titled "Infrared Photodissociation Spectroscopy of Fluoride Anion Hexafluoroisopropanol Complexes: Solvation-suppressed Proton Transfer" presents an interesting work on ion-molecule interactions. The work appears to be very basic, but the results presented are relevant for a better understanding of the interactions of ionic systems, such as halides, hydroxides, etc., with simple polar protic solvents. The experimental results using a cryogenically cooled ion trap triple mass spectrometer are sound. The theoretical methods used are in support of the experimental observations. These experiments are not so common. Hence, the results presented in the manuscript are

important for the broad readership of the community of physical chemistry and chemical physics in general and for the readers of J. Phys. Chem., in specific. The paper is overall well written, but there is scope for improvement in the presentation of the results: A concise and focused presentation of the introduction and discussion of the results would strengthen the overall quality of the manuscript. Hence, the manuscript may not be accepted in the present form. The authors are suggested to consider the following points:

1. There are a few general typographical errors in various parts of the manuscript. Careful proofreading is needed.

2. Proper descriptions of the Figures, Tables, etc. (i) Description of the Figure 1 in the text mentioned “spectral region from 950 to 3800 cm-1”; (ii) Difficult to identify the peak positions from the Figure as the values on start and end point of the X axis are not marked; (iii) Functional and basis set may be mentioned on Figure2 and so.

3. Further support is needed to justify the origin of the three weaker peaks in the vicinity of the strong OH stretching mode to Fermi resonance (Page 6, lines 50 onwards).

4. The relative intensity of the OH stretching mode is low in [F, (HFIP)3]- compared to the other two species with N = 2 and 1, whereas it is very close to that of [OH, HFIP]-. In [OH, HFIP]-, the OH stretching mode is for the HFIP. The authors did not discuss the spectral signature of the other OH oscillator, not even in ESI (3.2). This would be interesting to discuss briefly.

5. The importance of Figure 4 in the context of the present study is unclear.

6. Authors are suggested to describe Figure 1 in detail. More information might be buried in those spectra: a-c, c-e etc.

7. What are the local minima corresponding to the structures present in Figure 2? This results would be useful to conclude the effect of microsolvation beyond n = 4.

Author's Response to Peer Review Comments:

Response to Reviewers

We thank the reviewers for their careful reading of our manuscript and appreciate their helpful comments, which we have considered in preparing the revised manuscript. Find below a pointby-point response to all of the points the reviewers raised.

Reviewer 1

Technical suggestions 3- The authors presented the DFT minimum-energy structures of [F, (HFIP)$_{1-3}$]$^-$ in Figure 2 and ESI. I understand that the ionic hydrogen bonds are caused by the strong donor ability of the HFIP molecule. This ability is ascribed to the fluorination of the methyl groups in iso-propanol (i-PrOH). Thus, if the structures and interaction energies of [F, (HFIP)$_{1-3}$]$^-$ are compared to those of [F, (i-PrOH)$_{1-3}$]$^-$, we can probably understand the donor ability of HFIP more quantitatively. The author already reported the comparative study of HFIP and i-PrOH for the other halide anion complexes in reference 22. If the authors feel that such consideration is necessary, I would encourage them to revise it.

We provide the IRPD spectrum of the binary fluoride-isopropanol complex, [F, *i*-PrOH]$^-$, in Fig. S8 of the ESI. The observed red-shift of 2256 cm^{-1} is similar to that observed for [F, H_2O]$^-$. We also compare the present results to those previously published for [F, $(H_2O)_{1-3}$]$^-$ in Table 2 and discuss these in the manuscript. We did not extend the present study to the larger complexes [F, (*i*-PrOH)$_{2,3}$]$^-$, since this was beyond the scope of this work, but expect the IRPD spectra of these to exhibit similar red-shifts to those observed for the corresponding water complexes.

Reviewer 2

1. There are a few general typographical errors in various parts of the manuscript. Careful proofreading is needed.

We have corrected all the typos we could identify and tried to improve the form as indicated in the highlighted version.

2. Proper descriptions of the Figures, Tables, etc. (i) Description of the Figure 1 in the text mentioned "spectral region from 950 to 3800 cm-1";

We rechecked all the figures and tables captions. We replaced "from 950 cm^{-1} to 3800 cm^{-1}" by "1000 cm^{-1} to 3350 cm^{-1}" in the text, in the abstract and updated the caption of Figure 1 accordingly.

(ii) Difficult to identify the peak positions from the Figure as the values on start and end point of the X axis are not marked;

We adapted the x-axis such that the end points coincide with a tick mark. We also added the sentence

"Peak positions and assignments are reported in Table S2 and spectral scans up to 4000 cm^{-1} of D_2-tagged $[HFIP_{-H}]^-$, $[F, HFIP]^-$, $[OH, HFIP]^-$ are shown in Figure S3 of the ESI." near the top of page 3.

(iii) Functional and basis set may be mentioned on Figure2 and so.

We added the computational method, where necessary.

3. Further support is needed to justify the origin of the three weaker peaks in the vicinity of the strong OH stretching mode to Fermi resonance (Page 6, lines 50 onwards).

Yes, we agree. The assignment of the three weaker peaks for the $[Cl, HFIP]^-$ spectrum has been discussed in detail in ref. 22. It is based on a comparison to the results from anharmonic calculations using a variational approach (DVR-FBR technique). Such advanced anharmonic calculations are beyond the scope of the present work. Our assignment for $[F, (HFIP)_3]^-$ is simply based on the similarity of the two spectra and is therefore only tentative in nature. We added the word "tentatively" (page 7 / top) to emphasize this.

4. The relative intensity of the OH stretching mode is low in [F, (HFIP)3]- compared to the other two species with N = 2 and 1, whereas it is very close to that of [OH, HFIP]-. In [OH, HFIP]-, the OH stretching mode is for the HFIP. The authors did not discuss the spectral signature of the other OH oscillator, not even in ESI (3.2). This would be interesting to discuss briefly.

The reviewer addresses two important points here:

1) Care should be taken when comparing intensities of IRPD spectra using different laser configurations, and we therefore refrain from discussing relative band intensities in detail in this first section. To address this issue, we added the following text to the methods section of ESI:

"The covered spectral range 950–4000 cm^{-1} requires the use of two different laser configurations, which differ in the laser beam/ion cloud overlap. This overlap difference is difficult to quantify and we therefore did not correct for this. Consequently, even though the signal is corrected for the wavelength-dependent laser pulse energy to obtain $\square_{IRPD}$, the individual spectra covering these two

spectral regions (950–2220 cm^{-1} and 2060–4000 cm^{-1}) may systematically differ in $\square_{\mathrm{IRPD}}$. This should be considered when comparing the relative
IRPD band intensities between the two spectral regions."

We also added a sentence to the caption of Figure 1:

"Note, each IRPD spectrum consists of two parts, separated by a gap at 2250 cm^{-1}, which were measured with different IR laser configurations, complicating the comparison of the relative intensities between the two parts."

2) Concerning the position and assignment of O-H stretching bands in the IRPD spectrum of [OH, HFIP]$^{-}$, we added the following text at the bottom of page 7.

"Moreover, the extent of charge transfer is largest in [OH, HFIP]$^{-}$, where the corresponding OH stretching feature involving HFIP's O-atom is shifted below the measurement window. Note, this system is better viewed as HO-H…[HFIP−H]$^{-}$ and consequently the band at 2632 cm^{-1} observed in Figure 1b is due to excitation of the hydrogen-bonded OH oscillator of the H_2O (and not of the HFIP) moiety. The corresponding free O-H oscillator is observed at 3699 cm^{-1} (see Figure S3 of the ESI)."

5. The importance of Figure 4 in the context of the present study is unclear.

We agree that Figure 4 does not contribute to the central goal of this study, and we therefore removed it.

6. Authors are suggested to describe Figure 1 in detail. More information might be buried in those spectra: a-c, c-e etc.

We agree that there is probably more information buried in the spectra a-e shown in Figure 1. However, the aim of this paper is not to unravel and assign these spectral details, which would most surely also require a more advanced computational treatment. This can and will be done in a subsequent spectroscopic study. In this communication, we focus on reporting on the characterization of ionic hydrogen bonds in these systems containing strong hydrogen bonds and how their nature changes as proton transfer proceeds. We use the IRPD spectra mainly to determine red-shifts. Since this also requires a knowledge of the complexes' structure, we describe the spectra in such detail that an unambiguous structural assignment is possible.

7. *What are the local minima corresponding to the structures present in Figure 2? This results would be useful to conclude the effect of microsolvation beyond n = 4.*

The local minima for complexes with more than one HFIP molecule are presented in Figures S4-S6 of the ESI.
